# Expression Profile of mRNAs and miRNAs Related to the Oxidative-Stress Phenomenon in the Ishikawa Cell Line Treated Either Cisplatin or Salinomycin

**DOI:** 10.3390/biomedicines10051190

**Published:** 2022-05-20

**Authors:** Szymon Januszyk, Paweł Mieszczański, Hubert Lurka, Dorota Sagan, Dariusz Boroń, Beniamin Oskar Grabarek

**Affiliations:** 1 ICZ Healthcare Hospital in Zywiec, 34-300 Zywiec, Poland; 2 Hospital of Ministry of Interior and Administration, 40-052 Katowice, Poland; pawelmieszczanski102@gmail.com; 3 Department of Histology, Cytophysiology and Embryology, Faculty of Medicine, University of Technology, Academia of Silesia in Katowice, 41-800 Zabrze, Poland; bejoint@gmail.com (H.L.); dariusz@boron.pl (D.B.); 4 Medical Center Dormed Medical SPA, 28-105 Busko-Zdroj, Poland; dorotasagan@gmail.com; 5 Department of Gynecology and Obstetrics with Gynecologic Oncology, Ludwik Rydygier Memorial Specialized Hospital, 31-826 Kraków, Poland; 6 Department of Gynecology and Obstetrics, TOMMED Specjalisci od Zdrowia, 40-662 Katowice, Poland; 7 Department of Gynecology and Obstetrics, Faculty of Medicine, University of Technology, Academia of Silesia in Katowice, 41-800 Zabrze, Poland; 8 Gyncentrum, Laboratory of Molecular Biology and Virology, 40-851 Katowice, Poland

**Keywords:** cisplatin, salinomycin, oxidative stress, mRNA, miRNA, cell line, endometrial cancer, supplementary molecular marker, inflammation

## Abstract

The oxidative stress phenomenon is a result of anticancer therapy. The aim of this study was the assessment of gene expression profile changes, and to determine the miRNAs regulating genes’ transcriptional activity in an Ishikawa endometrial cancer culture exposed to cisplatin or salinomycin, compared to a control culture. The molecular analysis comprised the microarray technique (mRNAs and micro RNA (miRNA), the real-time quantitative reverse transcription reaction (RTqPCR), enzyme-linked immunosorbent assay (ELISA) reactions, and Western blot. NR4A2, MAP3K8, ICAM1, IL21, CXCL8, CCL7, and SLC7A11 were statistically significantly differentiated depending not only on time, but also on the drug used in the experiment. The conducted assessment indicated that the strongest links were between NR4A2 and hsa-miR-30a-5p and has-miR-302e, MAP3K8 and hsa-miR-144-3p, CXCL8 and hsa-miR-140-3p, and SLC7A11 and hsa-miR-144-3p. The obtained results suggest that four mRNAs—NR4A2, MAP3K8, CXCL8 and SLC7A11—and four miRNAs—hsa-miR-30a-5p, hsa-miR-302e, hsa-miR-144-3p and hsa-miR-140-3—changed their expressions regardless of the chemotherapeutic agent used, which suggests the possibility of their use in monitoring the severity of oxidative stress in endometrial cancer. However, considering the results at both the mRNA and the protein level, it is most likely that the expressions of NR4A2, MAP3K8, CXCL8 and SLC7A11 are regulated by miRNA molecules as well as other epigenetic mechanisms.

## 1. Introduction

In 2018, over 380,000 new cases of endometrial cancer were registered [1]. The frequency at which the cancer occurs is increasing year on year, and it is affecting women who are younger than in previous years. However, it continues to mainly occur in older, post-menopausal women, with only 4% of all patients being under 40 [2]. The reason for the increase in morbidity is the obesity epidemic and the hyperinsulinemia that results [3]. According to the World Health Organization (WHO), close to 40% of endometrial cancer cases are related to being overweight [4]. The other cases, however, are related to high blood pressure, excessive exposure to estrogens, type II diabetes, as well as tamoxifen usage, which is a synthetic, non-steroidal compound with an anti-estrogenic effect that is used in adjuvant therapy after primary therapy for breast cancer, or the treatment of advanced metastatic breast cancer [5]. More than 1 in 10 women diagnosed with endometrial cancer are at an advanced stage of the disease. However, endometrial cancer is most often diagnosed in grade G1 of histopathological differentiation. The standard treatment of the disease at a highly advanced stage is mainly based on cytoreduction surgery, followed by adjuvant therapy in the form of brachytherapy and/or radiotherapy, and in more advanced cases, chemotherapy and hormone therapy [6,7]. For younger women, in order to protect fertility and prevent illness, preventive treatment is recommended. Lifestyle changes, such as a healthy diet or physical activity, can lower the risk of the cancer occurring, as well as preventing relapses [3]. In in vitro studies, the following endometrial cancer cell lines are commonly utilized: Ishikawa (corresponds to grade G1 and type I endometrial cancer); HEC-1-A and HEC-1-B (correspond to grade G2 and type II endometrial cancer); KLE (corresponds to grade G3 and type II endometrial cancer) [8]. Salinomycin is an antibiotic that was invented in the twentieth century by Miyazaki et al. [9]. The first findings regarding the effectiveness of salinomycin in cancers characterized by a high risk of drug-resistance were described by Naujokat et al. In this work, the case of an 82-year-old vulvar cancer patient was described, who was treated with salinomycin, with a satisfactory clinical response being obtained [10]. There is, however, a lack of research regarding the influence of salinomycin in the context of endometrial cancer. Cisplatin is an inorganic compound containing a heavy metal (platinum), and it has been used since the 1970s in neoplastic diseases (molar mass 300.05 g/mol) [11]. In accordance with the recommendations of the Polish Society of Oncological Gynecology (PTGO), set out in 2017, in the case of endometrial cancer, cisplatin is used at a concentration of 60 mg/m^2^ in adjuvant therapy [12]. The term “oxidative stress” refers to the imbalance between oxidizing and reducing molecules, the latter of which there are fewer, resulting in the inability to fight reactive intermediates such as peroxides or free radicals, or repair the damage they cause [13].

In the physiological state, free radicals, defined as molecules or molecule fragments with one or more unpaired electrons in their atomic or molecular orbit, are produced in humans and other aerobic organisms [14]. However, in oxidative stress, free radicals can lead to negative effects such as structural damage or the breakage of a strand of deoxyribonucleic acid (DNA) [15]. This damage is mainly caused by reactive oxygen species (ROS), which are free radicals derived from oxygen [14]. In humans, oxidative stress has been noted in, among others, neoplastic processes [16], Alzheimer’s disease [17], Parkinson’s disease [18], and atherosclerosis [19]. On the other hand, there is also a beneficial role played by free radicals. The immune system uses oxidants to kill pathogens; phagocytes produce both ROS and reactive nitrogen species [20]. Furthermore, they play an important role in the regulation of gene expression, protein phosphorylation processes, or in influencing calcium concentration in cells [21].

It should also be noted that the oxidative stress phenomenon is a positive result of anticancer therapy. So far, knowledge about the influence of cisplatin and salinomycin on oxidative stress in endometrial cancer is fragmentary. Therefore, given that cisplatin is used in the treatment of endometrial cancer, and salinomycin is used in ovarian cancer, it seems that to determine the applicability of either drug in endometrial cancer, it is important to determine their influences on the phenomenon of oxidative stress [22]. Yu et al. assessed the influence of cisplatin on head and neck squamous cell carcinoma (HNSCC). These researchers noted that cisplatin halts the proliferation of cancer cells. Oxidative stress related to the administration of this chemotherapeutic agent induces changes in three catabolic pathways, glycolysis, the pentose phosphate pathway, and the citric acid cycle, highlighting that changes in lactate concentration can be utilized to determine sensitivity to cisplatin [23]. Additionally, the oxidative stress induced by cisplatin is the cause of nephrotoxicity arising during therapy with this drug, thus more and more research is concentrating on finding effective, natural antioxidants, such as hesperetin or resveratrol [24,25]. Sani et al. assessed the effect of cisplatin on oxidative stress in the induction of acute kidney injury. These researchers demonstrated that cisplatin activates oxidative stress by activating FasL/Fas-dependent oxidative renal tubular cell death [26]. Additionally, the study by Martins et al. showed that the toxicity of cisplatin towards nephrons and hepatocytes occurs as a result of increased oxidative stress in cells, and their death occurs primarily through the mitochondrial apoptotic pathway. This study was carried out on an animal model (Wistar rats) [27]. Additionally, Pratibha et al. indicated that cisplatin induces oxidative stress, the consequence of which is increased lipid peroxidation in the treated tissue of rats, as well as the lowered concentration of glutathione and glutathione reductase [28].

Salinomycin induces oxidative stress through the inhibition of the I and II complexes of the respiratory chain, decreasing the potential of the mitochondrial membrane. This in turn influences the activation of cascades dependent on the AMP-activated protein kinase (AMPK) and mTOR, which is expected in the context of cancer therapy [29]. Salinomycin significantly increases the production of ROS and 8-hydroxy-2′-deoxyguanosine (8-OH-dG), as well as lipid peroxidation. On the molecular level, salinomycin contributes to a reduction in the expression of nuclear factor erythroid 2-related factor 2 (NRF2), heme oxygenase-1, Nicotinamide adenine dinucleotides phosphate (NAD(P)H) quinone dehydrogenase 1 and glutamate-cysteine ligase catalytic subunit [30,31]. NRF2 plays a key role in maintaining the antioxidant potential of a cell, and a decrease in this protein’s expression confirms the anticancer qualities of salinomycin [32]. The research of Ketol et al., performed on the prostate cancer cell line, showed that salinomycin induces oxidative stress by reducing the antioxidant capacity of cells [33]. In turn, the observations of Kwang-Yiun et al. indicate that salinomycin induces the apoptosis of tumor cells by increasing the intracellular concentration of ROS, reducing the mitochondrial membrane potential. These observations indicate that salinomycin promotes apoptosis via a mitochondrial-dependent pathway [34]. Research also indicates that the induction of oxidative stress by anticancer drugs may also be associated with the unfavorable phenomenon of drug resistance in endometrial cancer cells during cisplatin therapy [35,36].

It has been proven that oxidative stress plays a highly significant role in damaging joint tissue, as well as in causing chronic inflammation in patients with rheumatoid arthritis (RA), which may lead to connective tissue degradation, and the deformation of joints and periarticular elements [37]. Tumor necrosis factor-alpha (TNF-α) is a strong indicator of oxidative stress. The molecular mechanism is connected to the fact that TNF-α, by interacting with specific receptors (TNFRI and TNFRII), activates signaling cascades dependent on nuclear factor kappa light chain-enhancer of activated B cells (NFkB), which, when acting as a transcription factor, enhances TNF-α biosynthesis. This is an example of a positive feedback loop [38,39]. In mice, which are homozygous for the deficiency of the cytoplasmic superoxide dismutase (Sod1) isoform, or heterozygous for the deficiency of the mitochondrial (Sod2) isoform, significant oxidative damage and spontaneously developing cancer were noted [40,41].

A significant role in the regulation of cell and metabolic processes, as well as gene expression, is played by epigenetic mechanisms, one of which is the RNA (RNAi) interference phenomenon, which involves 19–25 nucleotide microRNA (miRNA). These also play a key role in the induction and development of inflammation and carcinogenesis. The expression of most miRNA in tumor tissue is lower than in normal tissue, which results from DNA methylation.

However, considering the context, activated signaling cascades probably support tumor progression, survival, metastasis, and epithelial–mesenchymal transition (EMT) [42]. Tumor-associated miRNAs function as oncogenes in many types of cancer, such as endometrial, breast, ovarian, and colon cancer [43]. In the case of endometrial cancer, it has been indicated that the overexpression of certain microRNAs, such as miR-423, miR-103, miR-205, miR-429, and miR-135a, is involved in the carcinogenesis, progression, and proliferation of new cells. It has also been found that selected microRNAs, such as miR-221, miR-193, miR-30c, and miR99b, work in the opposite manner, inhibiting metastasis and uncontrolled tumor growth [44]. An important advantage related to using miRNAs as supplementary molecular markers is their tissue specificity and resistance to RNase, as well as the possibility of detecting changes in their expression using basic techniques of molecular biology [45].

The aim of this study was to assess variances in the expressions of genes related to oxidative stress, and the miRNAs regulating their transcriptional activity, in an Ishikawa cell line endometrial cancer culture exposed to cisplatin or salinomycin, compared to a control culture.

## 2. Results

### 2.1. The Results of Cytotoxicity Assay

**Figure 1:**
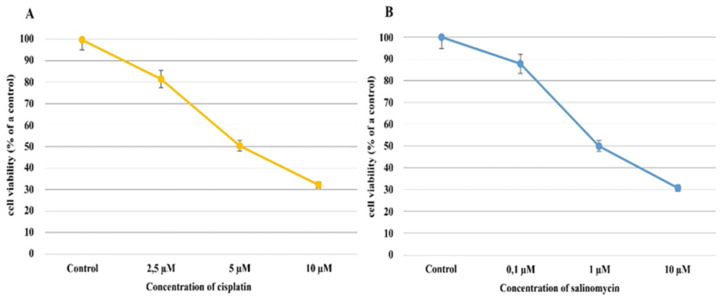
The results of the cytotoxicity assay of cisplatin (**A**) and salinomycin (**B**) on Ishikawa cell viability.

In the Ishikawa cell line exposed to cisplatin, regardless of the concentration of cisplatin, the percentage of viable cells decreased compared to the control culture. When cisplatin, at a 2.5 µM concentration, was added to the culture medium, the percentage of viable cells decreased by approximately 20% compared to a control culture (81.64% ± 1.76% mean ± SD; *p* = 0.001); furthermore, a drug concentration of 5 µM caused the percentage of viable cells to decrease by approximately 50% (IC50; 50.42% ± 1.38% mean ± SD *p* = 0.0002). In turn, when 10 µM of cisplatin was applied, the percentage of viable cells dropped to 32.23% ± 0.70% (mean ± SD) (*p* < 0.00001; Figure 1A).

Similar to the cisplatin, when the Ishikawa cell line was exposed to salinomycin, a decrease in the percentage of viable cells compared to the control culture was observed, regardless of salinomycin concentration. When salinomycin was added to the culture’s medium, at a concentration of 0.1 µM, the viable cell percentage decreased by approximately 20%, in comparison to the control culture (87.99% ± 0.09% (mean ± SD); *p* = 0.001), whereas at a concentration of 1 µM, a decrease in the percentage of viable cells of approximately 50% was observed (IC50; 49.99% ± 0.16%; *p* = 0.0002).

According to these results, 1 µM of salinomycin and 5 µM of cisplatin were selected as the average inhibitory concentrations (IC50s). The percentages of viable and dead endometrial cancer cells were almost equal, at around 50% (Figure 1).

### 2.2. The Level of ROS in Ishikawa Cells Treated with Cisplatin or Salinomycin

Based on the analysis of the induction of ROS formation under the influence of selected drugs to which endometrial cancer cells were exposed, it can be observed that both cisplatin and salinomycin induce the formation of ROS. This effect is dependent on the exposure time of the cells to the drug before adding DHE to the culture and on the chemotherapeutic agent itself (Table 1; *p* < 0.05).

### 2.3. The Results of the Microarray Analysis

Out of the 22.277 mRNAs currently on the HG-U133_A2 microarray slide, 3881 are connected to oxidative stress. The one-way ANOVA conducted in the first stage of the analysis indicated that 1452 mRNAs significantly differentiated the culture with cisplatin, compared to the control (*p* < 0.05), while 1402 mRNAs significantly differentiated the culture containing salinomycin (*p* < 0.05).

In total, 384 mRNAs were common between the Ishikawa cell lines exposed to cisplatin and salinomycin. A list of all the mRNAs (significantly differentiated) is presented in the Supplementary Material (Tables S1 and S2).

Tukey’s post hoc test (*p* < 0.05) was then performed for the 384 mRNAs mentioned above. The numbers of mRNAs differentiating the Ishikawa cell culture from the control when exposed to cisplatin, at different exposure times, are as follows: for the 12 h incubation time H_12 vs. C—172 mRNAs; for the 24 h incubation time H_24 vs. C—116 mRNAs; for the 48 h incubation time H_48 vs. C—96 mRNAs. Furthermore, 10 mRNAs—*NR4A2*, *MAP3K8*, *ICAM1*, *DUSP4*, *ADRA2A*, *IL21*, *CXCL8*, *CCL7*, *SLC7A11* and *PDGFA*—differentiated the culture containing the drug from the control, regardless of the incubation period (*p* < 0.05).

In contrast, in the Ishikawa endometrial cancer cell line exposed to salinomycin, the numbers of mRNAs differentiating the cell culture treated with the drug from the control culture, for individual exposure times, are as follows: for the 12 h incubation time H_12 vs. C—142 mRNAs; for the 24 h incubation time H_24 vs. C—136 mRNAs; for the 24 h incubation time H_48 vs. C—106 mRNAs. Moreover, 12 mRNAs—*NR4A2*, *MAP3K8*, *ICAM1*, *PLK3*, *HSPA2*, *INHBB*, *IL21*, *CXCL8*, *CCL7*, *SLC7A11*, *CCL20* and *ANGPTL4*—differentiated the culture containing the drug from the control, regardless of the incubation period (*p* < 0.05).

It was observed that *NR4A2*, *MAP3K8*, *ICAM1*, *IL21*, *CXCL8*, *CCL7*, and *SLC7A11* were statistically significantly differentiated depending not only on time, but also on the drug used in the experiment.

Although mRNA *TNF-α*, *NRF2*, *HIF1A*, and *HIF3A* were not common genes, considering their significant role in oxidative stress [23,24], we decided to analyze changes in their expression patterns under cisplatin vs. salinomycin treatment. Changes in the expression profiles of these gene, compared with the control, are presented in Table 2 (*p* < 0.05).

### 2.4. The Results of the RTqPCR

The data from the microarray were validated using a RTqPCR reaction.

**Figure 2:**
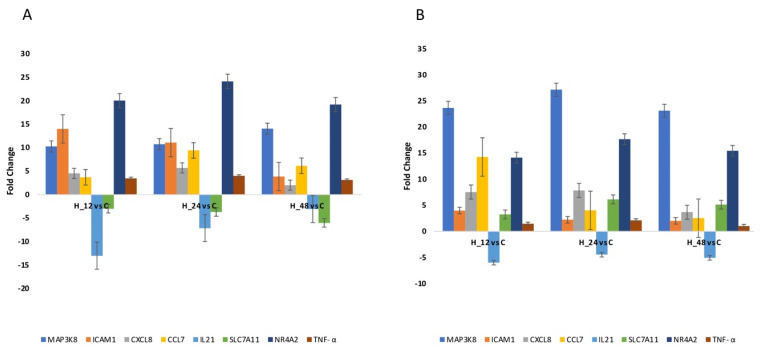
Changes in the expression patterns of genes related to oxidative stress in an Ishikawa cell culture exposed to cisplatin (**A**) and salinomycin (**B**), in comparison to the control obtained via RTqPCR (*p <* 0.05). (+)—overexpression in comparison to the control; (−)—downregulated in comparison to the control; C—control; H_12, H_24, H_48—periods of exposure to cisplatin or salinomycin; NR4A2—nuclear receptor subfamily 4 group A member 2; MAP3K8—mitogen-activated protein kinase 8; ICAM1—intercellular adhesion molecule 1; IL21—interleukin 21; CXCL8—C-X-C motif chemokine ligand 8; CCL7—C-C motif chemokine ligand 7; SLC7A11—solute carrier family 7 member 11.

Quantitative RTqPCR was performed to either confirm or eliminate the mRNA expression profiles derived via microarray analysis. The same direction of change in expression was noted for all genes assessed, regardless of the method utilized (Figure 2; *p* < 0.05). The results of the one-way ANOVA and post hoc Tukey’s test applied to the RTqPCR results are presented in Table 3.

### 2.5. Expression Pattern of Selected miRNAs

In the final stage of the molecular analysis performed on the transcriptome level, we indicated miRNAs that could be engaged in the regulation of *NR4A2*, *MAP3K8*, *ICAM1*, *IL21*, *CXCL8*, *CCL7*, and *SLC7A11*, assuming that the value of the predicted target had a prediction score > 80, as recommended [20].

The assessment indicated the strongest connections between *NR4A2* and hsa-miR-30a-5p and hsa-miR-302e, *MAP3K8* and hsa-miR-144-3p, *CXCL8* and hsa-miR-140-3p, and *SLC7A11* and hsa-miR-144-3p. It was observed that hsa-miR-144-3p can regulate the expressions of *NR4A2* and SLC7A11 (Table 4; *p* < 0.05).

### 2.6. Concentrations of NR4A2, MAP3K8, CXCL8 and SLC7A11 Determined via ELISA Assay

**Figure 3:**
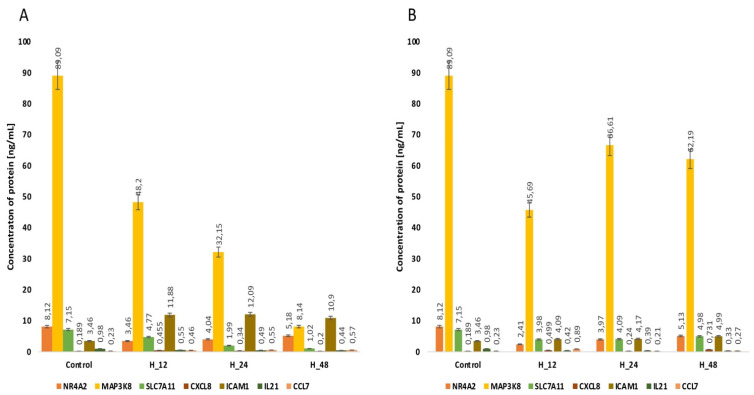
Variances in the concentration of the proteins NR4A2, MAP3K8, CXCL8 and SLC7A11 determined via ELISA assay in the Ishikawa cell line regardless of the time of incubation of endometrial cancer cells with either cisplatin (**A**) or salinomycin (**B**), in comparison with the control. C—control; H_12, H_24, H_48—periods of exposure to cisplatin or salinomycin; NR4A2—nuclear receptor subfamily 4 group A member 2; MAP3K8—mitogen-activated protein kinase kinase 8; CXCL8—C-X-C motif chemokine ligand 8; SLC7A11—solute carrier family 7 member 11; *p*—*p*-value.

Because miRNAs are expression-regulatory molecules at the post-transcriptional level, we evaluated changes in the concentrations of NR4A2, MAP3K8, CXCL8 and SLC7A11 proteins with an ELISA assay. This stage of our analysis allows the establishment of a more precise linkage between the selected mRNAs and miRNAs. For NR4A2, regardless of the drug and the time for which endometrial cells are exposed to it, we noted an increase in the concentration of this protein compared to the control. In turn, for CXCL8, we observed reductions in protein concentration in both cisplatin- and salinomycin-exposed endometrial cancer cultures. However, for MAP3K8 and SLC7A11, we found a different direction of change in the concentration profiles of these proteins, depending on the drug used (Figure 3, Table 5, *p <* 0.05).

### 2.7. Concentration of NR4A2, MAP3K8, CXCL8 and SLC7A11 Determined via Western Blot

**Figure 4:**
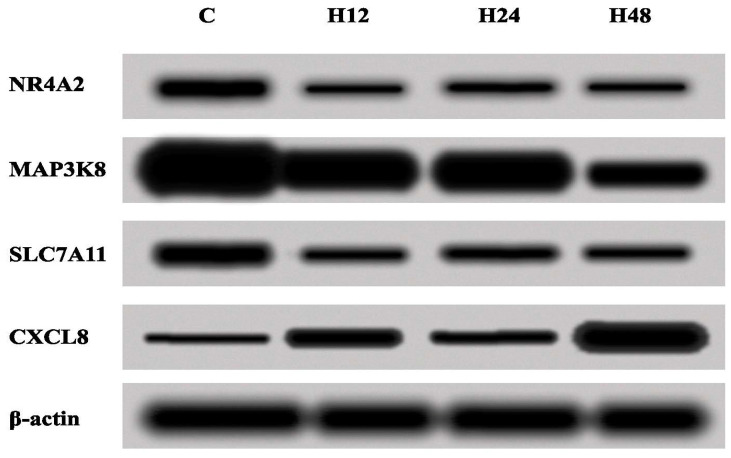
Expressions of NR4A2, MAP3K8, CXCL8 and SLC7A11 in the Ishikawa cell line treated with either cisplatin or salinomycin and a control culture, obtained via the Western blot technique. C—control; H_12, H_24, H_48—periods of exposure to cisplatin or salinomycin; NR4A2—nuclear receptor subfamily 4 group A member 2; MAP3K8—mitogen-activated protein kinase kinase 8; CXCL8—C-X-C motif chemokine ligand 8; SLC7A11—solute carrier family 7 member 11.

Next, the changes in the expression of NR4A2, MAP3K8, CXCL8 and SLC7A11 were assessed by the Western blot method, in order to validate the results obtained with the ELISA test. On the basis of the obtained Western blot results, changes in the concentrations of the proteins assessed were confirmed (Figure 4).

### 2.8. Summarizing the Changes in the Expression of the Selected mRNA-miRNA-Proteins

In the last stage, we showed the relationship between the expression of mRNA, miRNA regulating each mRNA and protein encoded by selected mRNA (Table 6).

In the Ishikawa culture exposed to cisplatin compared to the control culture, we observed that the overexpression of *MAP3K8* mRNA and *CXCL8* mRNA is accompanied by an increase in the expression of miRNAs potentially regulating their expression. However, on the protein level, the concentration of MAP3K8 is lower in the culture with cisplatin compared to the control, and the concentration of the CXCL8 protein is higher in the culture with the drug compared to the control. In turn, for *NR4A2*, we observed an increase in expression at the mRNA level, which was accompanied by a decrease in the expression of miRNAs regulating its expression and proteins. On the other hand, for *SLC7A11*, we noted a reduction in mRNA and protein expression with simultaneous overexpression of the miRNAs regulating its expression.

On the other hand, in the Ishikawa culture exposed to salinomycin, compared to the control culture, we found *NR4A2*, *MAP3K8*, *SLC7A11* mRNA overexpression with simultaneous silencing of the miRNAs potentially involved in the regulation of the indicated mRNAs and the proteins they encode. In turn, for *CXCL8*, the reduction in its expression at the mRNA level was accompanied by overexpression of the miRNAs regulating its expression and of the protein encoded by this gene.

### 2.9. Results of the Overrepresentation Test

The performed overrepresentation test showed that salinomycin significantly statistically influences a greater number of biological processes involving genes associated with the phenomenon of oxidative stress differentiating the culture with the drug compared to the control culture (15 vs. 8; Appendix A). Additionally, the number of activated signaling pathways under the influence of salinomycin is significantly greater than under the influence of cisplatin (11 vs. 4; Appendix B).

The analysis showed that the developmental potential of genes is related to immune response, DNA repair and intracellular signal transduction activities (Appendix A), while signaling pathways are directly related to inflammation, interleukin, angiogenesis, and B and T cell activation (Appendix B).

On the other hand, with regard to four genes differentiating the culture of endometrial cancer cells, regardless of the drug used for stimulation, compared to the control, we noted that the *MAP3K8* gene is associated with only one signaling pathway, i.e., gonado-tropin-releasing hormone receptor pathway. In turn, *CXCL8* mRNA is associated with five biological processes—inflammatory response, the antimicrobial humoral immune response mediated by antimicrobial peptide, the cellular response to lipopolysaccharide, granulocyte chemotaxis, neutrophil migration, and the cytokine-mediated signaling pathway—and three signaling pathways—the interleukin signaling-pathway, the CCKR8-signaling pathway and the inflammation signaling pathway.

The obtained results indicate which biological processes and signaling pathways in Ishikawa culture exposed to cisplatin or salinomycin are overrepresentative, which confirms that the mechanism of cisplatin and salinomycin displacement at the molecular level is related to genes related to oxidative stress.

## 3. Discussion

Oxidative stress occurs under both physiological and pathological conditions. Under normal conditions, as a result of metabolic processes taking place in the cell, which in turn result from antioxidant metabolization, ROS and free radicals are formed, which are strong modulators of signaling pathways [46,47,48,49]. Furthermore, ROS contribute to the damage of deoxyribonucleic acid (DNA) molecules, consequently leading to the formation of 8-OH deoxyguanosine adducts (8-OHdG), and this results in an increased risk of neoplastic mutagenesis [50]. Moreover, 8-OHdG can induce GC-to-TA base pair changes after DNA replication, inducing mutagenesis. Therefore, maintaining balance and homeostasis is important [51].

Therefore, considering the significant role played by oxidative stress in carcinogenesis and the response to anticancer therapy, in this study, we assessed the influence of cisplatin and salinomycin on the mRNA and miRNA transcriptomes of the Ishikawa endometrial cancer cell line.

PANTHER analysis of target mRNAs was performed to better understand the function of differentially expressed transcripts related to the oxidative stress phenomenon. The analysis showed that developmental potential of genes related to immune response, DNA repair and intracellular signal transduction activities as well as signaling pathways directly related to inflammation, interleukin, angiogenesis, and B and T cell activation, which is essential from the point of view of the mechanism of action of either cisplatin or salinomycin on cancer cells. Moreover, it confirms the complexity of the oxidative stress phenomenon and its direct relation to immunological processes. In addition, we confirmed that cisplatin and salinomycin added to the Ishikawa lineage of endometrial cancer cultures induce ROS formation.

The obtained results confirm that both cisplatin and salinomycin influence changes in the expression profiles of protein-encoding genes connected to oxidative stress in endometrial cancer cells in vitro. The statistical analysis of the microarray profile indicated that the transcripts *NR4A2*, *MAP3K8*, *CXCL8*, and *SLC7A11* differentiate the Ishikawa cell culture, regardless of both the time of exposition of the cells to drugs and the chemotherapeutic agents used to stimulate the cells. Under the influence of both cisplatin and salinomycin, *NR4A2*, *MAP3K8*, and *CXCL8* are overexpressed, and the direction of change in transcriptional activity for SLC7A11 differs depending on the drug used (cisplatin—silencing of expression; salinomycin—overexpression). The results of changes in the expression pattern of SLC7A11 in the Ishikawa culture, under the influence of either cisplatin or salinomycin, are intriguing. Huang et al. determined the overexpression of this gene in colorectal adenocarcinoma, esophageal squamous cell carcinoma, lung squamous cell carcinoma, rectum adenocarcinoma, and uterine corpus endometrial carcinoma [52]. On the other hand, Yu et al. indicated that an increase in the expression of SLC7A11 leads to a decrease in the concentration of enzymes involved in lipid peroxidation, and thus does not lead to acute kidney injury (AKI), which is a common side effect of cisplatin [53]. A reduction in the expression of SLC7A11 leads to a reduction in the concentration of glutathione, as well as cystine depletion, which results in increased lipid peroxidation and excessive protein degradation [54,55,56]. In addition, Sun et al. observed that under the influence of resveratrol, a strong antioxidant, the expressions of Nrf2 and SLC7A11 increase in a microglia cell line [57]. Therefore, as salinomycin caused the silencing of SLC7A11 in an endometrial cancer culture, the drug may induce tumor cell death through ferroptosis. This programmed cell death process differs from apoptosis, necrosis, and autophagy, and is mainly associated with the accumulation of ROS and lipid peroxidation in the cell [58,59].

The NR4A2 gene is a transcriptional factor belonging to the nuclear receptor superfamily, and involves three receptors, NR4A1–3, which mainly participate in the regulation of the cell cycle and the apoptosis, induction, and development of the inflammatory process or carcinogenesis [60]. Studies in recent years have indicated that the discussed gene and the protein it encodes affect the differentiation of the lymphocyte subpopulation from Th0 through to Th17, and is a critical regulator of lymphocytes, macrophages, and even fibroblasts [61]. Furthermore, an increase in NR4A2 expression may be characteristic of diseases whose etiology depends on Th17 lymphocytes [62]. Nonetheless, the role of the NR4A1 gene in carcinogenesis is ambiguous. It was indicated that in the case of lymphomas, pharmacotherapy aimed at increasing the expression of NR4A1 and NR4A3 is justified. In turn, the overexpression of NR4A1 and NR4A2 in solid tumors is an unfavorable prognostic marker, and therapy should be targeted at decreasing their expression [63]. On the other hand, Beard et al. highlighted that NR4A superfamily members are factors that inhibit tumor suppressor signaling [64], while Shigeishi et al. noted that these receptors are anti-apoptotic factors of neoplastic cells [65]—this, however, is not confirmed by our observations. The second gene differentiating the culture exposed to the drug from the control is MAP3K8. Lee et al. indicated that an overexpression of the discussed gene in invasive squamous cell carcinoma is linked to the progression of neoplastic changes [66]. Furthermore, Alves et al. highlighted that the overexpression of MAP3K8 occurs in 30% of endometrial cancer cases [67]. It should be remembered that MAP3K8 is a member of the ERK mitogen-activated protein kinase (MAPK) signaling cascade, against which anticancer therapy is targeted, and which is one of the several pathways associated with cisplatin resistance [68]. On the other hand, Zhang et al.’s ovarian cancer culture was treated with CD105 siRNA and also indicated an overexpression of MAP3K8, which is in this case a favorable result of cell transfection, and the inhibition of CD105 expression may be useful in the treatment of ovarian cancer [69]. Additionally, the results of the third transcript whose overexpression we determined, CXCL8, are different than expected, as an increase in its expression has been noted in many types of cancer [70,71].

Furthermore, considering our results and those of others, MAP3K8 and CXCL8 overexpression in an Ishikawa line endometrial cancer cell culture under the influence of cisplatin or salinomycin is most likely the result of the influence of these drugs on the cell cycle, and the established cancer cell homeostasis. It should also be remembered that our study was conducted under in vitro conditions.

Next, we decided to evaluate whether miRNAs are engaged in the regulation of the transcriptional activity of selected transcripts related to oxidative stress in an endometrial cancer cell culture exposed to cisplatin or salinomycin, compared to a control culture. The conducted predictive assessment indicated that the expressions of genes encoding proteins related to oxidative stress depended most closely on five miRNAs, regardless of the drug the endometrial cancer cells were incubated with. Wang et al. noted the overexpression of hsa-miR-30a-5p in glioblastoma samples, compared to normal tissue, highlighting the possibility of using this molecule as a supplementary diagnostic molecular marker in this type of cancer [72]. Świtlik et al. also observed an increased expression of hsa-miR-30a-5p in non-small cell lung cancer (NLSC) compared to normal lung tissue [73]. Interesting in the context of the study by Świtlik et al. [74], and particularly in the context of our research, are the observations of Zhu et al., who indicated a decrease in hsa-mir-30a-5p expression in NLSC samples compared to normal tissue [75]. Thus, it seems that the expression of miRNA molecules is not only tissue-specific, but also dependent on biological context [76,77], as is the case for transforming growth factor-beta (TGFβ) [78]. Attention should also be given to the important role of hsa-miR-30a-5p in the progression of ovarian cancer, indicating that an increased expression of this molecule is a prognostic marker of ovarian cancer development and correlates with disease-free survival [79,80]. Additionally, Han et al. indicated a connection between hsa-miR-30a-5p expression and DNA-methyltransferase isoform 1 (DNMT), and demonstrated the importance of this enzyme in the induction of cisplatin resistance in ovarian cancer cells [81,82]. The obtained research results suggest that hsa-miR-30a-5p may be a potential cancer therapy target [83], and that the complex influence of epigenetic mechanisms on the expression profiles of genes encoding proteins may be key in the carcinogenesis process. The second miRNA differentiating the culture with the drug from the control, and which potentially regulates the expression of the mRNA NR4A2, is hsa-miR-302e, which belongs to the miR-302 family, and is involved in the developmental processes of nerve cells [84,85,86,87]. Yang et al. [88] indicated that hsa-miR-302e is a negative expression regulator of the ORX1 gene related to oxidative stress. A decrease in the expression level of this gene leads to an increase in the number of ROS, consequently leading to the activation of apoptosis and necrosis pathways [87]. Our observations indicate that both cisplatin and salinomycin cause a reduction in the expression of hsa-miR-302e, which is accompanied by an increase in the transcriptional activity of the gene NR4A2. Chen et al. stated that these changes in the hsa-miR-302e expression profile can be used to assess the sensitivity of lung cancer cells to radiotherapy [88]. In our research, we also indicated that hsa-miR-144-3p can regulate the expression of MAP3K8 and SLC7A11; under the influence of cisplatin, hsa-miR-144-3p is overexpressed compared to the control, whereas salinomycin leads to the silencing of the expression of this miRNA. Turut et al. [89] exposed ovarian cancer cells (OVCAR-3 and SKOV-3) to anti-mir-144-3p for 36 h. They noted that the expression of this miRNA was accompanied by an increase in the expression of cyclooxygenase isoform 2 (COX-2), C-X-C motif chemokine receptor 4 (CXCR4), C-X-C motif chemokine ligand 12 (CXCL12), vascular endothelial growth factor (VEGF), caspase-3, BAX, and Bcl-2. Thus, the inflammatory process is intensified, the apoptosis of neoplastic cells is reduced, and the tumor progressed [90]. On the other hand, Chen et al. indicated that the overexpression of hsa-miR-144-3p in the case of cholangiocarcinoma (CHOL) is an unfavorable prognostic factor [91]. Furthermore, Wang et al. indicated that hsa-miR-144-3p promotes the proliferation of osteosarcoma cells [92]. Therefore, taking into consideration the contradictory observations presented regarding the meaning of hsa-miR-144-3p expression in particular types of cancer, alongside our results, including those on the cytotoxicity of cisplatin and salinomycin against Ishikawa line endometrial cancer cells, it should be assumed that the observed changes result from other mechanisms of the analyzed chemotherapeutic agents. Nonetheless, further research is required to determine the expression profiles of genes coding anti- and proapoptotic proteins. The final culture differentiating miRNA is has-miR-140-3p. This is the first study in which the aforementioned miRNA molecule was overexpressed (see Kapodistrias et al. [93]). The discrepancies resulting from the miRNAs’ expression in the Ishikawa cell culture treated with either cisplatin or salinomycin in comparison to a control culture were observed for hsa-miR-144-3p, which can potentially regulate the expression of *MAP3K8* and *SLC7A11*.

The opposite expression pattern of a given mRNA or miRNA in the culture of the Ishikawa line treated with cisplatin or salinomycin is not surprising, because a single miRNA can act both in up- and downregulation, and likewise a single specific mRNA could encounter both regulation directions based on the specific conditions and factors (another drug used to stimulate cells). Therefore, the direction of shift in mRNA and miRNA expression is specific to the type of cell, the factors to which the cell is exposed, and its condition. It has been confirmed that miRNA acting at the post-transcriptional level can both increase the concentration of the protein encoded by the gene and also act as a suppressor [94,95,96].

In the last step of our work, we evaluated variances in the concentrations of NR4A2, MAP3K8, CXCL8 and SLC7A11 proteins with an ELISA assay and Western blot. This stage of our analysis allows us to establish a more precise linkage between the selected mRNAs and miRNAs, because miRNAs are expression-regulatory molecules at the post-transcriptional level. The obtained protein concentration results were confirmed by the ELISA test by means of the Western blot technique.

However, our results for the relationship between mRNA-miRNA-protein expression may seem controversial. In the case of Ishikawa endometrial cancer cultures exposed to cisplatin, it appears that in the case of MAP3K8 and SLC7A11, the miRNAs regulating their expression act as expression suppressors at the PTGS level. In contrast, in the case of CXCL8, miRNA appears to be a molecule that enhances the expression of this gene, resulting in the overexpression of the CXCL8 protein in the cisplatin-treated culture compared to the control. On the other hand, in the case of NR4A2, we noted mRNA overexpression, with simultaneous silencing of the miRNA regulating its expression and proteins. Thus, it seems reasonable to conclude that, at least in the regulation of the expression of this gene, at the level of PTGS, another epigenetic mechanism is involved.

On the other hand, in the case of the culture of the Ishikawa line exposed to salinomycin, in the case of NR4A2, MAP3K8, and SLC7A11, we observed overexpression at the mRNA level, while for miRNA and proteins, we found a reduction in expression. This suggests that not only miRNAs are involved in the regulation of expression at the post-transcriptional level. Therefore, the participation of anti-microRNA molecules, as well as alternative splicing, the incorrect transport of mature mRNA from the cell nucleus to the cytoplasm where translation takes place, and the inhibition of the activities of other proteins, are also factors that determine the effective translation of these proteins. In turn, in the case of CXCL8, miRNAs involved in the regulation of this gene most likely act as enhancers of post-transcriptional expression [97,98].

In our work, we also presented a microarray profile of the expression of genes described in the literature as being closely related to the phenomenon of oxidative stress, i.e., *TNF-α*, *NRF1*, *HIF1A*, *HIF3A* [23,24], although these were not transcripts differentiating cultures treated with a drug from to a control culture.

We noted a decrease in the transcriptional activity of *NRF1*, *HIF1A*, and *HIF3A* in the culture exposed to the drugs cisplatin or salinomycin compared to the control. Zhihong et al. also found decreased expression of *HIF1A* encoding a protein involved in the glycolysis pathway in cisplatin-sensitive ovarian cancer cell lines. They found that *HIF1A* is a promising molecular marker for the assessment of the sensitivity of cancer cells to platinum compounds, as well as silencing its expression is a factor sensitizing cells to an anti-cancer drug [99]. Additionally, Song et al. emphasized that HIF factors may be a promising therapeutic target [100]. Similarly, Gao et al. confirmed that *NRF1* overexpression is an unfavorable prognostic factor in breast cancer, associated with cell resistance to cisplatin [101]. Thus, our results indicate that Ishikawa endometrial cancer cells are sensitive to cisplatin as well as salinomycin.

Nevertheless, it should be noted that the analysis of the interaction between mRNA and miRNA was determined at the predictive level, and was also included in it, in line with the recommendations. Therefore, it cannot be ruled out that in fact the remaining miRNAs for which the conditions specified in the recommendations have not been met have a greater than expected effect on the regulation of the expression of proteins encoded by the selected genes. In addition, it should be kept in mind that despite the enormous development of molecular biology, there are still many things that have not been fully explained. Therefore, it may be necessary to perform advanced bioinformatics analyses as well as the transfection of endometrial cancer cells exposed to a given drug with small interfering RNA (siRNA) in order to determine the actual interaction between mRNA and protein.

The strengths of our research include the use of modern molecular biology methods, such as microarray analysis, the semi-quantitative results of which were validated using the quantitative RTqPCR method, as well as the mRNA and miRNA transcriptome analysis of cancer cells under the influence of cisplatin and salinomycin. In addition, we evaluated changes in the concentrations of the selected proteins via two independent methods: ELISA and Western blot. Of course, our research has some limitations. First of all, the analysis should be extended to other endometrial cancer cell lines [102], given that HEC-1-A and HEC-1-B correspond to histological grade 2-G2 and type II, and KLE corresponds to histological grade 3-G3 and type II [8,103]; we should also incorporate in vivo analysis. Secondly, it is important to determine the possibility of regulating the expression of oxidative stress genes via other epigenetic mechanisms, such as DNA methylation, or the post-translational modification of histone proteins.

In summary, the results obtained here are ambiguous, and need to be extended; however, they constitute a significant starting point for further research, and provide a better understanding of the oxidative stress phenomenon in endometrial cancer under the influence of chemotherapeutic agents.

## 4. Materials and Methods

This section builds upon our previous works [104,105].

### 4.1. Ishikawa Endometrial Cancer Cell Culture

In this study, for each biological replicate, three technical replicates were performed. Ishikawa cell line endometrial cancer cell cultures (European Collection of Authenticated Cell Cultures; ECACC 99040201) were treated with different cisplatin concentrations—2.5 µM; 5 µM; and 10 µM—or different salinomycin concentrations—0.1 µM; 1 µM; 10 and µM. The cells were exposed to these drugs for 12, 24, and 48 h periods. Untreated cells formed the control cell culture in this experiment. The minimum essential medium (MEM; Sigma-Aldrich, St. Louis, MO, USA) was used for this cell line, supplemented with 2 mM of glutamine, 1% non-essential amino acids (NEAA, Sigma-Aldrich, St. Louis, MO, USA), and 5% fetal bovine serum (FBS, Sigma-Aldrich, St. Louis, MO, USA), in accordance with the manufacturer’s protocol. The cells were incubated at a constant temperature of 37 °C with a 5% CO_2_-enriched atmosphere (Direct Heat CO_2_; Thermo Scientific, Waltham, MA, USA).

### 4.2. Sulforhodamine B Cytotoxicity Test

The sulforhodamine B sodium salt assay (Sigma-Aldrich, St. Louis, MO, USA, Catalog number 3520-42-1) was used to determine the cytotoxic properties of the drugs. In the first stage of this analysis, Ishikawa cells in the logarithmic growth phase were seeded into a 24-well plate at a concentration of 20,000 cells/^2^ mL medium per well, followed by incubation at 37 °C with a 5% CO_2_ (Direct Heat CO_2_; Thermo Scientific, Waltham, MA, USA)-enriched atmosphere for 24 h. Next, Ishikawa cells were exposed to cisplatin in a concentration range of 2.5 to 10 µM, or salinomycin in a concentration range of 0.1 µg/mL to 100 µg/mL. Untreated cells constituted the control. Readings were taken at a wavelength of 490–530 nm.

The absorbance results obtained from the control culture were defined as the base, described as 100%. This allowed us to identify the concentrations of cisplatin and salinomycin that inhibited the proliferation of cells by 50% (IC50). This concentration was used for the exposition of endometrial cancer cells to the drugs for 12-, 24-, and 48-h periods.

### 4.3. Ribonucleic Acid Extraction

Firstly, the TRIzol reagent (Invitrogen Life Technologies, Carlsbad, CA, USA, Catalog number 15596026) was used, according to the manufacturer’s protocol, to extract the total ribonucleic acid (RNA) from the cell culture treated with either cisplatin or salinomycin, as well as from a control culture.

Secondly, to purify the RNA isolates, the RNeasy Mini Kit (QIAGEN, Hilden, Germany, Catalog number 74104) and DNase I enzyme (Fermentas International Inc., Burlington, ON, Canada, Catalog number 18047019) were used. Extracts were diluted in 20 µL of sterile water, then frozen at −70 °C until molecular analysis was performed.

### 4.4. Quantitative and Qualitative Evaluation of RNA Extracts

Electrophoresis on a 1% agarose gel stained with 0.5 mg/mL ethidium bromide (Sigma Aldrich, St. Louis, MO, USA) was performed in order to qualitatively evaluate the total extracted RNA. Placing the electropherogram image in the light of the UV transilluminator resulted in the observation of two bands corresponding to the 28 S rRNA and 18 S rRNA fractions.

Spectrophotometry was used to determine the concentration and purity of the RNA extracts, assuming that 1 OD260 = 40 µg RNA in 1 mL of extract. Absorbance increments at other wavelengths indicated contamination (230 nm—carbohydrate contamination, ethanol residues, EDTA; 280 nm—protein; 320 nm—cellular particles, degradation of the genetic material in the sample). The purity of the RNA extracts was evaluated based on the A260/A280 ratio value (standard 1.8–2.0).

### 4.5. Microarray Profile of Oxidative Stress-Related Genes

Changes in the transcriptional activity of mRNAs related to oxidative stress in Ishikawa cells exposed to cisplatin or salinomycin, compared with the control culture, were analyzed, according to the manufacturer’s recommendation, using the HG-U 133_A2 microarray (Affymetrix, Santa Clara, CA, USA), the GeneChip™ 3′ IVT PLUS Reagent Kit, and the GeneChip™ HT 3′ IVT PLUS Reagent Kit (Affymetrix, Santa Clara, CA, USA, Catalog number 902416). Three technical repetitions were performed for each biological replicate.

Out of the 22,277 mRNA probes, 3881 mRNAs were related to oxidative stress. This was determined on the basis of the “Affymetrix NetAffx Analysis Center database” after entering the query: “oxidative stress” (https://www.affymetrix.com/analysis/netaffx/showresults.affx; accessed on 17 November 2021). The microarray experiment involved a few steps. The first step involved the synthesis of double-stranded cDNA, using the GeneChip 30IVT Express Kit (2 h at 420 °C). Secondly, 20 μL of Second Strand Master Mix was added, and incubated for 1 h at 160 °C, and then again at 650 °C for 10 min. The third stage of the experiment was biotinylated aRNA synthesis. To achieve this, 30 µL of IVTMaster Mix for cDNA was added into the mixture and then incubated for 16 h at 40 °C. Next, the cDNA was fragmented using a matrix fragmentation buffer for 35 min at 940 °C. Finally, the GeneChip Hybridization, Wash, and Stain Kit was used to prepare the hybridization of the mixture. The Affymetrix Gene Array Scanner 3000 7G and GeneChip^®^ Command Console^®^ Software were utilized to analyze the fluorescence and intensity (Affymetrix, Santa Clara, CA, USA).

### 4.6. Microarray Profile of miRNAs Related to the Oxidative Stress and Potential Influence on the Expression of Analyzed Genes

The commercially available GeneChip miRNA 2.0 Array (Affymetrix, Santa Clara, CA, USA) was used to determine the microarray profiles of miRNAs according to the manufacturer’s recommendations. Three technical repetitions were performed for each biological replicate.

To determine which of the differentiating miRNAs of the Ishikawa cell line, combined with cisplatin or salinomycin, affected the transcriptional activity of the differentiating mRNAs, compared to the control culture, we used the miRDB tool (http://mirdb.org/; accessed on 17 November 2021). According to the miRDB database, “This is an online database for miRNA target prediction and functional annotations. All the targets in miRDB were predicted by a bioinformatics tool, MirTarget, which was developed by analyzing thousands of miRNA-target interactions from high-throughput sequencing experiments. Common features associated with miRNA binding and target downregulation have been identified and used to predict miRNA targets with machine learning methods” [106].

### 4.7. Real-Time Quantitative Reverse Transcription Reaction

The reverse-transcription quantitative polymerase chain reaction (RTqPCR) was performed to validate the results obtained with the semi-quantitative microarray technique.

We used the SensiFast™ SYBR No-ROX One-Step Kit (Bioline, London, UK), and β-actin (ACTB) as the endogenous control. The volume of the reaction mixture was 50 µL. The thermal profile of the reaction was as follows: reverse transcription (45 °C, 10 min); activation of the polymerase (95 °C, 2 min); 40 cycles including denaturation (95 °C, 5 s); annealing (60 °C, 10 s); and elongation (72 °C, 5 s). The primer sequence is presented in Table 3. Analysis was performed with an Opticon™ DNA Engine Sequence Detector (MJ Research Inc., Watertown, MA, USA) using the SYBR Green Quantitect RT-PCR Kit (Qiagen, Valencia, CA, USA). The nucleotide sequence of primers used in RTqPCR was presented in Table 7.

Changes in the expression patterns of selected genes obtained via RTqPCR are shown as a fold change in gene expression in comparison to a control.

### 4.8. Enzyme-Linked Immunosorbent Assay Reaction

The last stage of this study involved assessing differences in the concentrations of NR4A2, MAP3K8, CXCL8, SLC7A11, ICAM1, IL21 and CCL7 via an enzyme-linked immunosorbent assay reaction (ELISA). We used commercially available kits, such as the beta-Thromboglobulin (beta-TG; CXCL8) ELISA Kit (MyBioSource, Inc., San Diego, CA, USA, 92195-3308; Catalog number MBS264511), the mitogen-activated protein kinase 8 ELISA Kit (MyBioSource, Inc., San Diego, CA, USA, 92195-3308; Catalog number MBS9325582), the Nuclear Receptor Related Protein 1 (NURR1; NR4A2) RTU ELISA Kit (MyBioSource, Inc., San Diego, CA, USA, 92195-3308; Catalog number MBS4501531), the cystine/glutamate transporter, SLC7A11, ELISA Kit (MyBioSource, Inc., San Diego, CA, USA, 92195-3308; Catalog number MBS1606069), the ICAM-1 (ICAM-1/CD54) ELISA Kit (MyBioSource, Inc., San Diego, CA, USA, 92195-3308; Catalog number MBS2515841), the IL21 ELISA Kit (MyBioSource, Inc. San Diego, CA, USA, 92195-3308; Catalog number MBS454439) and the Human CCL7/MCP-3 ELISA Kit (MyBioSource, Inc. San Diego, CA, USA, 92195-3308; Catalog number MBS1753914), in accordance with the manufacturers’ recommendations. Three technical repetitions were performed for each biological replicate.

### 4.9. Western Blot Analysis

The last stage of our experiment involved assessing the concentrations of NR4A2, MAP3K8, CXCL8 and SLC7A11 via Western blot according to the manufacturer’s protocol. M-Per mammalian protein extraction reagent (Thermofisher, Scientific, Waltham, MA 02451, United States, Catalog numer 78501) and protease inhibitor cocktail for mammalian tissues (Sigma Aldrcih, St. Louis, MO, USA, Catalog number P8340) were used to extract proteins from Ishikawa endometrial carcinoma cells exposed to cisplatin or salinomycin, as well as from a control culture. Protein electrophoresis was performed on 12.5% sodium dodecyl–sulfate–polyacrylamide gel electrophoresis (SDS-PAGE) gel. After electrophoresis was completed, the proteins separated from the gel were dry transferred using two flat electrodes, between which the gel and a polyvinylidenefluoride (PVDF) membrane (without transfer buffer) were placed. A blocking buffer, 5% skim milk in TPBS (PBS + 0.1% Tween 20), was used to block non-specific sites on the membrane to which proteins are not bound. Blocking was performed at +40 °C overnight. After washing the membrane, it was incubated with primary antibodies directed specifically against the protein of interest: anti-NR4A2 antibody (Sigma Aldrich, St. Louis, MO, USA, Catalog number AV3273; 1:1000), anti-MAP3K8 antibody (Sigma Aldrich, St. Louis, MO, USA, Catalog number SAB4500411; 1:500), anti-CXCL8 antibody (Sigma Aldrich, St. Louis, MO, USA; 1:1000), or anti- SLC7A11 antibody (Sigma Aldrich, St. Louis, MO, USA; 1:1000). Subsequently, secondary anti-HRP antibodies, conjugated with horseradish peroxidase at a dilution of 1:5000, were used. Densitometric analysis was performed using Kodak MI 4.5SE software. The results obtained for the evaluated proteins were normalized against β-actin and are presented as the relative optical density.

### 4.10. Determination of the Level of Reactive Oxygen Species (Method with Dihydroethidine)

The level of superoxide anion radical (O^2^ • ─) was determined by the microplate spectrofluorimetric method with dihydroethidine (DCF). DCF is considered as the most specific and stable fluorescent probe for monitoring O^2^ • ─ generation. It is retained by the cell and can also be used to stain fixed cells and in in vivo tests. DHE is characterized by blue fluorescence (λex = 355 nm, λem = 430 nm). As a result of the reaction between DHE and O^2^ • ─, the highly specific product 2-hydroxyethidine (2-OH-E +) was formed, which after intercalation to DNA showed a strong red fluorescence (λex = 460 nm, λem = 640 nm). After incubation with cisplatin or salinomycin at the IC50, medium was removed, the cell monolayer in each well was washed twice with HBSS (140 mM NaCl, 5 mM KCl, 0.8 mM MgCl_2_, 1.8 mM CaCl_2_, 1 mM Na_2_HPO_4_, 10 mM HEPES and 1% glucosa) and added to each well with 50 μL of dihydroethidine solution (final concentration 615 μmol/L). Cells were incubated with the probe for 20 min under conditions appropriate to the cell line (37 °C), and then fluorescence was measured at 0, 30, 60, 90 and 120 min. The measurement was performed at two wavelengths of excitation and fluorescence emission: λex = 360 nm/λem = 420 nm (for dihydroethidine) and λex = 360 nm/λem = 640 nm (for ethidium). The fluorescence ratio 640 nm/420 nm was calculated. This ratio for the controls was set to 0% and the fluorescence of the samples was converted into ∆f relative to the control.

### 4.11. Overrepresentative Test

In the last stage of our analysis, we performed a binomial overrepresentation test with Bonferoni correction using the Protein Analysis Through Evolutionary Relationship (PANTHER) tool (*p* < 0.05) [107] in order to identify those signaling pathways and biological processes that can be most strongly altered as a consequence of the observed transcriptome changes (based on the number of genes involved in these pathways and processes).

## 5. Conclusions

Due to the fragmentary nature of the knowledge on the influence of cisplatin and salinomycin on oxidative stress in endometrial cancer, our molecular analysis is important in terms of cognition. We have shown that cisplatin and salinomycin exert a cytotoxic effect on endometrial cancer cells, as well as modulating the process of oxidative stress. The obtained results suggest that four mRNAs—NR4A2, MAP3K8, CXCL8 and SLC7A11—and four miRNAs—hsa-miR-30a-5p, hsa-miR-302e, hsa-miR-144-3p and hsa-miR-140-3—change their expressions regardless of the chemotherapeutic agent used, which suggests the possibility of their use in monitoring the severity of oxidative stress in endometrial cancer. Therefore, it cannot be ruled out that the remaining miRNAs for which the conditions specified in the recommendations have not been met have a greater than expected effect on the regulation of the expression of proteins encoded by the selected genes. In addition, it should be kept in mind that despite the enormous development of molecular biology, there are still many things that have not been fully explained. Therefore, it may be necessary to perform advanced bioinformatics analyses as well as the transfection of endometrial cancer cells exposed to a given drug with small interfering RNA (siRNA) in order to determine the actual interaction between mRNA and protein.

## Figures and Tables

**Table 1:** Changes in DCF fluorescence in Ishikawa cultures exposed to cisplatin or salinomycin compared to untreated cultures.

Drug	Time [Hours]	Time with DCF					
		Control	30 min	60 min	90 min	120 min	*p* < 0.05
cisplatin	0	100%	98.12 ± 9.31	94.16 ± 7.52	90.34 ± 12.94	81.58 ± 10.73	0.8930
	12	100%	127.45 ± 8.65	132.09 ± 12.76	145.89 ± 15.11	123.09 ± 17.34	0.0421
	24	100%	131.76 ± 21.09	156.99 ± 24.09	150.18 ± 12.99	141.92 ± 16.51	0.0034
	48	100%	198.34 ± 32.98	211.13 ± 10.31	232.09 ± 15.01	198.33 ± 32.54	0.0221
salinomycin	0	100%	101.98 ± 8.91	95.12 ± 8.11	91.15 ± 8.96	82.99 ± 11.59	0.8712
	12	100%	187.09 ± 12.98	287.13 ± 12.01	265.02 ± 45.17	212.71 ± 18.45	0.0039
	24	100%	268.11 ± 11.09	264.12 ± 18.49	276.12 ± 19.99	270.12 ± 31.05	0.0031
	48	100%	278.12 ± 13.87	256.99 ± 17.58	280.19 ± 19.98	289.19 ± 21.04	0.0023

DCF, dihydroethidine; *p*—statistically significant differences determined by post–hoc Tukey’s test.

**Table 2:** Expression profiles of genes differentiating the Ishikawa cell line, regardless of the time of incubation with either cisplatin or salinomycin, in comparison with the control.

mRNA	ID	Ishikawa Cell Line Treated with Cisplatin			Ishikawa Cell Line Treated with Salinomycin		
		H_12 vs. C	H_24 vs. C	H_48 vs. C	H_12 vs. C	H_24 vs. C	H_48 vs. C
*NR4A2*	204621_s_at	+18.41 (*p* = 0.0000)	+24.11 (*p* = 0.0000)	+20.07 (*p* = 0.0000)	+14.14 (*p* = 0.0000)	+16.99 (*p* = 0.0000)	+15.45 (*p* = 0.0000)
	204622_x_at	+17.52 (*p* = 0.0000)	+23.69 (*p* = 0.0000)	+21.11 (*p* = 0.0000)	+14.88 (*p* = 0.0000)	+16.75 (*p* = 0.0000)	+15.96 (*p* = 0.0000)
	216248_s_at	+18.36 (*p* = 0.0000)	+22.89 (*p* = 0.0000)	+20.18 (*p* = 0.0000)	+14.78 (*p* = 0.0000)	+16.99 (*p* = 0.0000)	+16.01 (*p* = 0.0000)
*MAP3K8*	205027_s_at	+10.11 (*p* = 0.0000)	+10.84 (*p* = 0.0000)	+14.11 (*p* = 0.0000)	+24.11 (*p* = 0.0000)	+27.14 (*p* = 0.0000)	+23.08 (*p* = 0.0000)
*ICAM1*	213191_at	+14.04 (*p* = 0.0000)	+12.01 (*p* = 0.0000)	+3.41 (*p* = 0.0089)	+3.99 (*p* = 0.0073)	+2.22 (*p* = 0.0123)	+2.01 (*p* = 0.0200)
*CXCL8*	202859_x_at	+4.14 (*p* = 0.0099)	+6.12 (*p* = 0.0077)	+2.01 (*p* = 0.0201)	+7.17 (*p* = 0.0044)	+7.14 (*p* = 0.041)	+3.00 (*p* = 0.0109)
	211506_s_at	+4.08 (*p* = 0.0012)	+6.22 (*p* = 0.0074)	+1.87 (*p* = 0.0418)	+7.14 (*p* = 0.0065)	+7.01 (*p* = 0.0066)	+3.21 (*p* = 0.00209)
*CCL7*	208075_s_at	+3.11 (*p* = 0.0121)	+9.41 (*p* = 0.0001)	+6.14 (*p* = 0.0009)	+12.01 (*p* = 0.0000)	+3.75 (*p* = 0.0230)	+3.01 (*p* = 0.0231)
*IL21*	221271_at	−12.44 (*p* = 0.0000)	−6.41 (*p* = 0.0077)	−3.07 (*p* = 0.0019)	−5.99 (*p* = 0.0010)	−4.47 (*p* = 0.0077)	−5.07 (*p* = 0.0021)
*SLC7A11*	207528_s_at	−2.99 (*p* = 0.0238)	−3.48 (*p* = 0.0099)	−5.17 (*p* = 0.0001)	+3.25 (*p* = 0.0088)	+6.14 (*p* = 0.0076)	+5.02 (*p* = 0.0028)
	209921_at	−2.98 (*p* = 0.0236)	−3.42 (*p* = 0.0096)	−5.11 (*p* = 0.0076)	+3.41 (*p* = 0.0232)	+6.11 (*p* = 0.0072)	+4.99 (*p* = 0.0034)
	217678_at	−3.04 (*p* = 0.0277)	−3.49 (*p* = 0.0211)	−5.09 (*p* = 0.0011)	+3.21 (*p* = 0.0199)	+6.17 (*p* = 0.0051)	+4.14 (*p* = 0.0077)
*TNF-α*	207113_s_at	+3.76 (*p* = 0.0131)	+4.07 (*p* = 0.0112)	+3.14 (*p* = 0.0520)	+1.54 (*p* = 0.0531)	+1.98 (*p* = 0.0502)	+1.11 (*p* = 0.0653)
*NRF1*	211280_s_at	−1.78 (*p* = 0.0570)	−1.99 (*p* = 0.0591)	−2.12 (*p* = 0.0499)	−1.98 (*p* = 0.0513)	−2.32 (*p* = 0.0517)	−1.97 (*p* = 0.0741)
	204651_at	−1.91 (*p* = 0.0528)	−2.02 (*p* = 0.0522)	−1.91 (*p* = 0.0513)	−2.12 (*p* = 0.0527)	−2.31 (*p* = 0.0510)	−2.01 (*p* = 0.0491)
	204652_s_at	−1.66 (*p* = 0.0615)	−1.74 (*p* = 0.0618)	−2.08 (*p* = 0.0518)	−2.13 (*p* = 0.0517)	−2.19 (*p* = 0.0516)	−1.71 (*p* = 0.0618)
*HIF1A*	200989_at	−2.89 (*p* = 0.0501)	−2.76 (*p* = 0.0501)	−2.98 (*p* = 0.0501)	−3.87 (*p* = 0.0456)	−3.90 (*p* = 0.0502)	−3.12 (*p* = 0.0501)
*HIF3A*	219319_at	−2.15 (*p* = 0.0542)	−2.87 (*p* = 0.0512)	−1.78 (*p* = 0.0541)	−2.19 (*p* = 0.0518)	−2.44 (*p* = 0.0510)	−2.12 (*p* = 0.0521)
	222123_s_at	−2.18 (*p* = 0.0541)	−2.91 (*p* = 0.0500)	−1.61 (*p* = 0.0608)	−2.13 (*p* = 0.0512)	−2.41 (*p* = 0.0519)	−1.99 (*p* = 0.0611)
	222124_s_at	−2.02 (*p* = 0.0503)	−2.32 (*p* = 0.0519)	−1.74 (*p* = 0.0614)	−2.19 (*p* = 0.0511)	−2.59 (*p* = 0.0516)	−2.07 (*p* = 0.0512)

(+)—overexpression in comparison to the control; (−)—downregulated in comparison to the control; C—control; H_12, H_24, H_48—periods of exposure to cisplatin or salinomycin; NR4A2—nuclear receptor subfamily 4 group A member 2; MAP3K8—mitogen-activated protein kinase 8; ICAM1—intercellular adhesion molecule 1; IL21—interleukin 21; CXCL8—C-X-C motif chemokine ligand 8; CCL7—C-C motif chemokine ligand 7; SLC7A11—solute carrier family 7 member 11; TNF-α—tumor necrosis factor alpha; NRF1, nuclear factor erythroid 2-related factor 1; HIF1A, Hypoxia Inducible Factor 1 Subunit Alpha; HIF3A, Hypoxia Inducible Factor 3 Subunit Alpha; *p*—*p*-value.

**Table 3:** Results of the one-way ANOVA and post hoc Tukey’s test on RTqPCR results.

mRNA	Ishikawa Cell Line Treated with Cisplatin			Ishikawa Cell Line Treated with Salinomycin		
	H_12 vs. C	H_24 vs. C	H_48 vs. C	H_12 vs. C	H_24 vs. C	H_48 vs. C
*NR4A2*	*p* = 0.0000	*p* = 0.0000	*p* = 0.0000	*p* = 0.0000	*p* = 0.0000	*p* = 0.0000
*MAP3K8*	*p* = 0.0000	*p* = 0.0000	*p* = 0.0000	*p* = 0.0000	*p* = 0.0000	*p* = 0.0000
*ICAM1*	*p* = 0.0000	*p* = 0.0000	*p* = 0.0086	*p* = 0.0071	*p* = 0.0130	*p* = 0.0204
*CXCL8*	*p* = 0.0099	*p* = 0.0072	*p* = 0.0199	*p* = 0.0054	*p* = 0.048	*p* = 0.0101
*CCL7*	*p* = 0. 0121	*p* = 0. 0002	*p* = 0. 0000	*p* = 0.0000	*p* = 0.0231	*p* = 0.0233
*IL21*	*p* = 0.0000	*p* = 0.0072	*p* = 0.0012	*p* = 0.0012	*p* = 0.0069	*p* = 0.00219
*SLC7A11*	*p* = 0.0238	*p* = 0.0091	*p* = 0.0001	*p* = 0.0083	*p* = 0.0071	*p* = 0.0024
*TNF-α*	*p* = 0.0026	*p* = 0.0015	*p* = 0.0034	*p* = 0.0269	*p* = 0.0328	*p* = 0.0692

C—control; H_12, H_24, H_48—periods of exposure to cisplatin or salinomycin; NR4A2—nuclear receptor subfamily 4 group A member 2; MAP3K8—mitogen-activated protein kinase 8; ICAM1—intercellular adhesion molecule 1; IL21—interleukin 21; CXCL8—C-X-C motif chemokine ligand 8; CCL7—C-C motif chemokine ligand 7; SLC7A11—solute carrier family 7 member 11; TNF- α—tumor necrosis factor alpha; *p*—*p*-value.

**Table 4:** Expression profiles of miR-30a-5p, miR-302e, miR-144-3p, and miR-140-3p in an endometrial cancer cell line exposed to cisplatin or salinomycin, compared to the control.

mRNA	miRNA	Target Score mRNA:miRNA	Ishikawa Cell Line Treated with Cisplatin			Ishikawa Cell Line Treated with Salinomycin		
			miRNA			miRNA		
			H_12 vs. C	H_24 vs. C	H_48 vs. C	H_12 vs. C	H_24 vs. C	H_48 vs. C
*NR4A2*	hsa-miR-30a-5p	88	−4.41 * (*p* = 0.0017)	−4.85 * (*p* = 0.0016)	−4.96 * (*p* = 0.0015)	−10.02 * (*p* = 0.0000)	−7.11 * (*p* = 0.0000)	−8.54 * (*p* = 0.0000)
	hsa-miR-302e	82	−12.01 * (*p* = 0.0000)	−11.41 * (*p* = 0.0000)	−3.41 * (*p* = 0.0065)	−2.01 * (*p* = 0.0072)	−3.44 * (*p* = 0.0063)	−3.84 * (*p* = 0.0062)
*MAP3K8*	hsa-miR-144-3p	90	+6.15 * (*p* = 0.0001)	+9.39 * (*p* = 0.0000)	+8.74 * (*p* = 0.0000)	−8.12 * (*p* = 0.0000)	−12.01 * (*p* = 0.0000)	−2.03 * (*p* = 0.0208)
*SLC7A11*		96						
*CXCL8*	hsa-miR-140-3p	98	+3.69 * (*p* = 0.0065)	+2.01 * (*p* = 0.0213)	+3.14 * (*p* = 0.0076)	+2.11 * (*p* = 0.0223)	+4.01 * (*p* = 0.0035)	+4.53 * (*p* = 0.0023)

(+)—overexpression in comparison to the control; (−)—downregulated in comparison to the control; C—control; H_12, H_24, H_48—periods of exposure to cisplatin or salinomycin; NR4A2—nuclear receptor subfamily 4 group A member 2; MAP3K8—mitogen-activated protein kinase kinase 8; CXCL8—C-X-C motif chemokine ligand 8; SLC7A11—solute carrier family 7 member 11; *—statistically significant differences vs. C (*p* < 0.05); *p*—*p*-value.

**Table 5:** Results of the one-way ANOVA and post hoc Tukey’s test for ELISA results.

Protein	Ishikawa Cell Line Treated with Cisplatin			Ishikawa Cell Line Treated with Salinomycin		
	H_12 vs. C	H_24 vs. C	H_48 vs. C	H_12 vs. C	H_24 vs. C	H_48 vs. C
NR4A2	*p* = 0.0000	*p* = 0.0000	*p* = 0.0000	*p* = 0.0000	*p* = 0.0000	*p* = 0.0000
MAP3K8	*p* = 0.0000	*p* = 0.0000	*p* = 0.0000	*p* = 0.0000	*p* = 0.0000	*p* = 0.0000
SLC7A11	*p* = 0.0000	*p* = 0.0000	*p* = 0.0000	*p* = 0.0000	*p* = 0.0000	*p* = 0.0000
CXCL8	*p* = 0.0000	*p* = 0.0000	*p* = 0.0000	*p* = 0.0000	*p* = 0.0000	*p* = 0.0000
ICAM1	*p* = 0.0000	*p* = 0.0000	*p* = 0.0000	*p =* 0.6145	*p =* 0.9676	*p =* 0.9182
IL21	*p* = 0.0441	*p* = 0.0408	*p* = 0.0398	*p* = 0.0312	*p* = 0.0241	*p* = 0.0198
CCL7	*p* = 0.0782	*p* = 0.0517	*p* = 0.0471	*p* = 0.00137	*p* =0.9841	*p* = 0.9136

C—control; H_12, H_24, H_48—periods of exposure to cisplatin or salinomycin; NR4A2—nuclear receptor subfamily 4 group A member 2; MAP3K8—mitogen-activated protein kinase kinase 8; CXCL8—C-X-C motif chemokine ligand 8; SLC7A11—solute carrier family 7 member 11; *p*—*p*-value.

**Table 6:** Summarizing the changes in the expression of the selected mRNA-miRNA-proteins.

Group	Ishikawa Cells Treated with Cisplatin in Comparison to a Control			Ishikawa Cells Treated with Salinomycin in Comparison to a Control		
Expression	mRNA	miRNA Related to mRNA	Protein	mRNA	miRNA Related to mRNA	Protein
*NR4A2*	up	down	down	up	down	down
*MAP3K8*	up	up	down	up	down	down
*SLC7A11*	down	up	down	up	down	down
*CXCL8*	up	up	up	down	up	up

(up)—overexpression in comparison to the control; (down)—downregulated in comparison to the control; NR4A2—nuclear receptor subfamily 4 group A member 2; MAP3K8—mitogen-activated protein kinase kinase 8; ICAM1—intercellular adhesion molecule 1; IL21—interleukin 21; CXCL8—C-X-C motif chemokine ligand 8; CCL7—C-C motif chemokine ligand 7; SLC7A11—solute carrier family 7 member 11.

**Table 7:** The sequence of primers used in RTqPCR.

mRNA	Nucleotide Sequence	
*NR4A2*	Forward	5′-TATATGATCGAGTAGAGGAAAACGT-3′
	Reverse	5′-TACGAATAAAATTAAACACAACGAA-3′
*MAP3K8*	Forward	5′-TCGTCGGATTTTAGTGGTTC-3′
	Reverse	5′-AAAAATTACATCTACGACCTTAACG-3′
*CXCL8*	Forward	5′-TCGTCGGATTTTAGTGGTTC-3′
	Reverse	5′-AAAAATTACATCTACGACCTTAACG-3′
*SLC7A11*	Forward	5′-TAGTTTGAAAGTAGAGGAAGATATCGA-3′
	Reverse	5′-TCTAACCATAATAAAAACACACGAA-3′

NR4A2, nuclear receptor subfamily 4 group A member 2; MAP3K8, mitogen-activated protein kinase 8; CXCL8, C-X-C motif chemokine ligand 8; SLC7A11, solute carrier family 7 member 11.

## Data Availability

The data used to support the findings of this study are included in the article. The data will not be shared due to third-party rights and commercial confidentiality.

## Supplementary Materials

The following supporting information can be downloaded at: https://www.mdpi.com/article/10.3390/biomedicines10051190/s1, Table S1: List of all the mRNAs (significantly differentiated) cell culture treated with cisplatin in comparison to a control culture; Table S2: List of all the mRNAs (significantly differentiated) cell culture treated with salinomycin in comparison to a control culture.

## Appendix A

**Table A1:** Biological processes modulated by genes related to the phenomenon of oxidative stress in Ishikawa cells exposed to cisplatin or salinomycin.

Group	Biological Process	Number of Gene	Fold Change	Symbol of the Gene	*p*-Value
Ishikawa cells treated with cisplatin	Positive regulation of T cell activation	6	12.77	*HLA-DQB1*, *HSPD1*, *HLA-DRB4*, *HLA-DRB5*, *HLA-DRB3*, *HLA-DRB1*	0.018
	Antigen processing and presentation	6	12.15	*HLA-DQB1*, *HLA-DRB4*, *MARCH8*, *HLA-DRB5*, *HLA-DRB3*, *HLA-DRB1*	0.023
	Cellular response to tumor necrosis factor	6	11.07	*CXCL1*, *CCL7*, *CCL5*, *IKBKB*, *CCL16*, *TNF*	0.014
	Leukocyte cell–cell adhesion	7	10.20	*HLA-DQB1*, *HSPD1*, *HLA-DRB4*, *HLA-DRB5*, *TNFRSF14*, *HLA-DRB3*, *HLA-DRB1*	0.014
	Negative regulation of intracellular signal transduction	9	7.95	*TSC1*, *DUSP3*, *HRH4*, *TNFAIP1*, *SRC*, *TNIP1*, *DUSP10*, *DUSP1*, *BCL2*	0.005
	Inflammatory response	101	5.97	*GGT5*, *CXCL1*, *CCL7*, *CCL5*, *CXCL9*, *MAPKAPK2*, *CXCL8*, *NLRP2*, *CCL18*, *IL37*	0.017
	DNA repair	13	2.37	*DCLRE1C*, *MSH2*, *TRRAP*, *OTUB1*, *CETN1*, *XPC*, *RAD50*, *GTF2H2C*, *RFC2*, *RAD51*, *DBRE*, *ERCC1*, *GTF2H2*	0.022
	Intracellular signal transduction	30	3.04	*TSC1*, *ARHGEF2*, *ADCYAP1R1*, *TRIM13*, *CXCL1*, *CCL7*, *DUSP3*, *TPD52L1*, *HRH4*, *TNFAIP1*, *MAPKAPK2*, *MAPK7*, *SRC*, *TNIP1*, *DUSP10*, *NOS3*, *DDIT3*, *CCL18*, *PIK3CD*, *DUSP1*, *AKT1*, *IKBKG*, *BCL2*, *PKD2*, *SH2B1*, *AKT2*, *HRH1*, *MAP2K1*, *PDPK1*	0.000
Ishikawa cells treated with salinomycin	Antimicrobial humoral immune response mediated by antimicrobial peptide	8	22.69	*S100A12*, *CXCL3*, *CAMP*, *CXCL8*, *CXCL5*, *PF4V1*, *DEFA6*, *REG3A*	0.000
	Cellular response to lipopolysaccharide	7	14.53	*CXCL3*, *CD86*, *CXCL8*, *CXCL5*, *PF4V1*, *DEFA6*, *LY86*	0.002
	Granulocyte chemotaxis	8	13.61	*CCL17*, *CXCL3*, *CCL7*, *CXCL8*, *CXCL5*, *CCL22*, *PF4V1*, *IL4*	0.001
	Regulation of lymphocyte mediated immunity	6	12.45	*KLRD1*, *MICB*, *C4BPA*, *IL4*, *MICA*, *KLRC3*	0.020
	Neutrophil migration	7	12.41	*CCL17*, *CXCL3*, *CCL7*, *CXCL8*, *CXCL5*, *CCL22*, *PF4V1*	0.004
	Receptor signaling pathway via JAK-STAT	6	11.60	*JAK2*, *INFA4*, *IL6R*, *IFNA8*, *IL15*, *IL4*	0.030
	Cytokine-mediated signaling pathway	17	9.84	*MPL*, *CCL17*, *JAK2*, *IFNA4*, *CXCL3*, *IL12B*, *IL5RA*, *CCL7*, *LILRA2*, *IL6R*, *IFNA8*, *LILRA1*, *CXCL8*, *CXCL5*, *OSMR*, *CCL22*, *PF4V1*	0.000
	Inflammatory response	14	8.57	*CCL17*, *CXCL3*, *NFKB2*, *TBXA2R*, *PTGER3*, *AOAH*, *CCL7*, *CXCL8*, *CXCL5*, *CCL22*, *PF4V1*, *TNFRSF1A*, *IL4*, *IL1RL2*	0.000
	Positive regulation of cell population proliferation	8	7.48	*CD38*, *CD86*, *IL6R*, *IL15*, *FGF7*, *IL4*, *REG3A*, *PTK2*	0.001
	Cell population proliferation	11	5.20	*IFNA4*, *CD38*, *CD86*, *IL6R*, *IFNA8*, *IL15*, *FGF7*, *IL4*, *REG3A*, *CDKN2D*, *PTK2*	0.16
	Cellular response to DNA damage stimulus	13	4.39	*WRN*, *RAD1*, *MSH3*, *OGG1*, *XPA*, *RECQL5*, *KIN*, *BCL2A1*, *BLM*, *MUTYH*, *RAD52*, *POLQ*, *CUL4B*	0.022
	Protein phosphorylation	18	3.62	*JAK2*, *IFNA4*, *PDK4*, *PAK2*, *LEFTY2*, *IL6R*, *TAB2*, *TPD52L1*, *IFNAB*, *TLK1*, *IL15*, *FGF7*, *IL4*, *PRKAA2*, *CDKN2D*, *PTK2*, *PPM1E*, *PRKCB*	0.007
	Intracellular signal transduction	26	2.70	*S100A12*, *CCL17*, *RAD1*, *JAK2*, *NFAT5*, *ADGRE2*, *NPRL2*, *PAK2*, *NFKB2*, *TBXA2R*, *ARHGEF2*, *RAPGEF4*, *PTGER3*, *CCL7*, *TPD52L1*, *PRKD1*, *ADORA2B*, *TLK1*, *DGKZ*, *RAP1B*, *BCL2A1*, *CCL22*, *BLNK*, *PRKAA2*, *NGFR*, *PRKCB*	0.010
	Regulation of molecular function	23	2.67	*S100A12*, *CCL7*, *STAC*, *ADGRE2*, *NFKB2*, *TBXA2R*, *RAPGEF4*, *PTGER3*, *CCL7*, *IL6R*, *TAB2*, *TPD52L1*, *ADORA2B*, *OASL*, *SERPINH1*, *CCL22*, *IL4*, *CRHBP*, *CASP8*, *CDKN2D*, *EPHA1*, *IL1RAP*, *PPM1E*	0.011
	Regulation of response to stimulus	29	2.49	*KLRD1*, *S100A12*, *CCL17*, *THEMIS2*, *IFNA4*, *ADGRE2*, *NPRL2*, *PAK2*, *LEFTY2*, *TBXA2R*, *ARHGEF2*, *PTGER3*, *MICB*, *AOAH*, *F2*, *CCL7*, *IL6R*, *C4BPA*, *TPD52L1*, *ADORA2B*, *IFNA8*, *IL15*, *CCL22*, *IL4*, *CRHBP*, *MICA*, *KLRC3*, *IL1RL2*, *LY86*	0.012

## Appendix B

**Table A2:** Signaling pathways modulated by genes related to the phenomenon of oxidative stress in Ishikawa cells exposed to cisplatin or salinomycin.

Group	Signaling Pathway	Number of Gene	Fold Change	Symbol of the Gene	*p*-Value
Ishikawa cells treated with cisplatin	Blood coagulation	9	16.65	*F12*, *F13B*, *F2*, *PROZ*, *GP5*, *F10*, *PROS1*, *FGA*, *F8*	0.000
	Interleukin signaling pathway	7	6.69	*ELK1*, *IL21*, *IL5RA*, *IL6R*, *CXCL8*, *IL15*, *IL4*	0.018
	CCKR8 signaling map	12	5.90	*ELK1*, *RYR2*, *JAK2*, *SCL18A2*, *PTPN11*, *CD38*, *PRKD1*, *MEF2C*, *CXCL8*, *CCK*, *PTK2*, *PRKC3*	0.000
	Inflammation mediated by chemokine and cytokine signaling pathway	17	5.54	*SOCS7*, *CAMK2A*, *JAK2*, *CXCR1*, *NFAT5*, *PAK2*, *NFKB2*, *CCL7*, *SOCS6*, *CXCL8*, *IL15*, *ITGAM*, *ITGA4*, *CCL22*, *PF4V1*, *ITGA9*, *PRKCB*	0.000
Ishikawa cells treated with salinomycin	Blood coagulation	11	13.71	*F12*, *F13B*, *F2*, *GP5*, *F10*, *PROS1*, *FGA*, *F8*, *KNG1*, *PLAU*	0.000
	Interferon-gamma signaling pathway	7	13.38	*SOCS6*, *JAK2*, *IFNGR1*, *PTPN11*, *MAPK11*, *SOCS7*, *MAPK14*	0.000
	CCKR8 signaling map	21	6.96	*ELK1*, *RYR2*, *ITGB1*, *BAX*, *JAK2*, *PTEN*, *SLC18A2*, *PTPN11*, *CD38*, *PRKD1*, *PRKCD*, *MEF2C*, *CXCL8*, *MAPK14*, *PXN*, *BRAF*, *CCK*, *PTK2*, *PP3CA*, *PRKCB*, *PLAU*	0.000
	VEGF signaling pathway	8	6.75	*PRKD1*, *PRKCD*, *MAPK14*, *PXN*, *BRAF*, *PTK*, *HSPB2*, *PRKCB*	0.000
	B-cell activation	8	6.46	*NFKB2*, *MAPK11*, *PRKCD*, *MAPK14*, *BLNK*, *BRAF*, *PPP3CA*, *PRKCB*	0.000
	Apoptosis signaling pathway	13	5.96	*AFT6B*, *HSPA5*, *BAX*, *NFKB2*, *HSPA1B*, *HSPA1A*, *PRKCD*, *FADD*, *TNFRSF1*, *CASP8*, *HSPA13*, *PRKCB*	0.000
	Inflammation mediated by chemokine and cytokine signaling pathway	25	5.49	*SOCS6*, *CXCR4*, *ITGB1*, *CAMK2A*, *JAK2*, *PTEN*, *CXCR1*, *IFNGR1*, *NFAT5*, *PAK2*, *NFKB2*, *MYH9*, *CCL7*, *SOCS7*, *PTAFR*, *CXCL8*, *ITGAM*, *IL15*, *MYLK*, *ITGA4*, *CCL22*, *PF4V1*, *ITGA9*, *BRAF*, *PRKCB*	0.000
	Interleukin signaling pathway	8	5.16	*ELK1*, *IL21*, *IL5RA*, *IL6R*, *CXCL8*, *IL15*, *IL4*, *BRAF*	0.030
	Parkinson disease	9	5.11	*ELK1*, *HSPA5*, *CUL1*, *HSPA1B*, *HCK*, *MAPK14*, *SNCA*, *HSPA13*	0.013
	T-cell activation	8	4.99	*PAK2*, *NFKB2*, *CD86*, *CD28*, *TRBV19*, *BRAF*, *TRBC2*, *PP3CA*	0.037
	Gonadotropin-releasing hormone receptor pathway	17	4.11	*ELK1*, *ITGB1*, *DRD2*, *PTGER3*, *HSPA1B*, *MAP3K8*, *MAPK11*, *HSPA1A*, *PRKCD*, *DGKZ*, *RAP1B*, *MAPK14*, *PXN*, *EGFR*, *PTK2*, *PPP3CA*, *PRKCB*	0.000
