# Supplementary material for: Expression Profile of mRNAs and miRNAs Related to the Oxidative-Stress Phenomenon in the Ishikawa Cell Line Treated Either Cisplatin or Salinomycin
Source: Biomedicines. 2022 May 20;10(5):1190. doi: 10.3390/biomedicines10051190 (PMC9138494; doi:10.3390/biomedicines10051190)
Supplement: Supplementary file 1 [file biomedicines-10-01190-s001.zip › table s1.pdf]

Table S1. List of all the mRNAs (significantly differentiated; $p < 0.05$) cell culture treated with cisplatin in comparison to a control culture.

H_12 vs C				H_24 vs C				H_48 vs C			
ID	Gene	Fold Change	up/down	ID	Gene	Fold Change	up/down	ID	Gene	Fold Change	up/down
222151_s_at	CEP63	-13.36	down	**202708_s_at**	HIST2H2BE	-12.54	down	**203151_at**	MAP1A	-9.77	down
221926_s_at	IL17RC	-12.06	down	**1053_at**	RFC2	-6.70	down	**205197_s_at**	ATP7A	-6.73	down
220451_s_at	BIRC7	-7.70	down	**205568_at**	AQP9	-6.33	down	**203148_s_at**	TRIM14	-6.51	down
221188_s_at	CIDEB	-7.39	down	**209460_at**	ABAT	-6.25	down	**205126_at**	VRK2	-5.30	down
220525_s_at	AUP1	-6.19	down	**209375_at**	XPC	-6.00	down	**207528_s_at**	SLC7A11	-5.17	down
200714_x_at	OS9	-5.86	down	**205567_at**	CHST1	-5.76	down	209921_at	SLC7A11	-5.11	down
201106_at	GPX4	-5.82	down	**202655_at**	MANF	-5.39	down	**205151_s_at**	TRIL	-4.61	down
200668_s_at	UBE2D3	-5.76	down	**209383_at**	DDIT3	-5.20	down	**200892_s_at**	TRA2B	-4.07	down
221556_at	CDC14B	-5.55	down	**202716_at**	PTPN1	-5.04	down	**205098_at**	CCR1	-3.81	down
203717_at	DPP4	-5.41	down	**202723_s_at**	FOXO1	-5.02	down	**203088_at**	FBLN5	-3.70	down
201498_at	USP7	-5.40	down	**202660_at**	ITPR2	-4.90	down	**203147_s_at**	TRIM14	-3.51	down
221552_at	ABHD6	-5.38	down	**202644_s_at**	TNFAIP3	-4.88	down	**201405_s_at**	COPS6	-3.49	down
200742_s_at	TPP1	-5.38	down	**209459_s_at**	ABAT	-4.87	down	**205141_at**	ANG	-3.44	down
201537_s_at	DUSP3	-5.34	down	**209364_at**	BAD	-3.71	down	**203149_at**	PVRL2	-3.36	down
221555_x_at	CDC14B	-5.27	down	**202652_at**	APBB1	-3.44	down	**203085_s_at**	TGFB1	-3.14	down
201107_s_at	THBS1	-5.26	down	**209361_s_at**	PCBP4	-2.60	down	**202859_x_at**	CXCL8	-3.10	down
201538_s_at	DUSP3	-5.23	down	**209368_at**	EPHX2	-2.47	down	**221271_at**	IL21	-3.07	down
201536_at	DUSP3	-5.11	down	**209357_at**	CITED2	-2.35	down	**203047_at**	STK10	-3.04	down
203688_at	PKD2	-4.80	down	**202642_s_at**	TRRAP	1.92	up	**205128_x_at**	PTGS1	-2.94	down
201272_at	AKR1B1	-4.74	down	**209404_s_at**	TMED7	1.94	up	**203050_at**	TP53BP1	-2.88	down
1431_at	20.00 CYP	-4.48	down	**209414_at**	FZR1	1.95	up	**205198_s_at**	ATP7A	-2.85	down

201208_s_at	TNFAIP1	-4.24	down	**205593_s_at**	PDE9A	2.19	up	**203089_s_at**	HTRA2	-2.83	down
200824_at	GSTP1	-4.19	down	**209453_at**	SLC9A1	2.23	up	**205189_s_at**	FANCC	-2.76	down
201164_s_at	PUM1	-4.00	down	**209349_at**	RAD50	2.24	up	**205099_s_at**	CCR1	-2.32	down
222233_s_at	DCLRE1C	-3.84	down	**209416_s_at**	FZR1	2.29	up	**205150_s_at**	TRIL	-2.18	down
200924_s_at	SLC3A2	-3.75	down	**209432_s_at**	CREB3	2.37	up	**203111_s_at**	PTK2B	1.70	up
200930_s_at	VCL	-3.55	down	**203888_at**	THBD	2.40	up	**205026_at**	STAT5B	1.77	up
201489_at	PPIF	-3.40	down	**209421_at**	MSH2	2.41	up	**205083_at**	AOX1	1.85	up
56748_at	TRIM10	-3.36	down	**209396_s_at**	CHI3L1	2.51	up	**201422_at**	IFI30	1.90	up
200810_s_at	CIRBP	-3.33	down	**209451_at**	TANK	2.58	up	**205158_at**	RNASE4	2.04	up
201188_s_at	ITPR3	-3.33	down	**209456_s_at**	FBXW11	2.61	up	**205050_s_at**	MAPK8IP 2	2.08	up
201161_s_at	YBX3	-3.11	down	**202637_s_at**	ICAM1	2.66	up	**203061_s_at**	1.00 MDC	2.24	up
201300_s_at	PRNP	-3.07	down	**205566_at**	ABHD2	2.66	up	**205040_at**	ORM1	2.25	up
221356_x_at	P2RX2	-3.05	down	**209417_s_at**	IFI35	2.66	up	**203081_at**	CTNNBIP 1	2.27	up
45288_at	ABHD6	-2.92	down	**203824_at**	TSPAN8	2.74	up	**205014_at**	FGFBP1	2.29	up
41858_at	PGAP2	-2.85	down	**207113_s_at**	TNF	2.81	up	**205022_s_at**	FOXN3	2.42	up
221169_s_at	HRH4	-2.84	down	**202672_s_at**	ATF3	2.81	up	**209020_at**	OSER1	2.48	up
201242_s_at	ATP1B1	-2.77	down	**209435_s_at**	ARHGEF2	2.82	up	**201415_at**	GSS	2.49	up
221372_s_at	P2RX2	-2.75	down	**202724_s_at**	FOXO1	2.97	up	**203040_s_at**	HMBS	2.50	up
200943_at	HMGN1	-2.71	down	**205550_s_at**	BRE	2.97	up	**203110_at**	PTK2B	2.53	up
203809_s_at	AKT2	-2.61	down	**203837_at**	MAP3K5	2.99	up	**203084_at**	TGFB1	2.59	up
203706_s_at	FZD7	-2.56	down	**209443_at**	SERPINA5	3.01	up	**203074_at**	ANXA8 /// ANXA8L1 /// LINC0084 2	2.69	up
201105_at	LGALS1	-2.27	down	**209407_s_at**	DEAF1	3.03	up	**205075_at**	SERPINF2	2.70	up
201286_at	SDC1	-2.25	down	**209415_at**	FZR1	3.06	up	**203109_at**	UBE2M	2.72	up

201187_s_at	ITPR3	-2.07	down	**207151_at**	ADCYAP1R 1	3.07	up	**205170_at**	STAT2	2.74	up
203705_s_at	FZD7	-2.05	down	**209406_at**	BAG2	3.08	up	**209029_at**	COPS7A	2.79	up
201293_x_at	PPIA	-1.97	down	**203900_at**	SZT2	3.11	up	**205033_s_at**	DEFA1 /// DEFA1B /// DEFA3	2.82	up
201437_s_at	EIF4E	1.79	up	**207160_at**	IL12A	3.14	up	**203052_at**	C2	2.82	up
221491_x_at	HLA-DQB1 /// HLA-DRB1 /// HLA-DRB2 /// HLA-DRB3 /// HLA-DRB4 /// HLA-DRB5 /// HLA-DRB6 /// HLA-DRB7 /// HLA-DRB8 /// LOC105369230	2.13	up	**205582_s_at**	GGT5	3.45	up	**205162_at**	ERCC8	2.85	up
221470_s_at	IL37	2.15	up	**202638_s_at**	ICAM1	3.51	up	**203041_s_at**	LAMP2	2.88	up
221406_s_at	MSH5-SAPCD1 /// SAPCD1	2.21	up	**203922_s_at**	CYBB	3.53	up	**203046_s_at**	TIMELESS	2.89	up

221518_s_at	USP47	2.24	up	**203868_s_at**	VCAM1	3.66	up	**205021_s_at**	FOXN3	2.93	up
221539_at	EIF4EBP1	2.26	up	**207209_at**	CETN1	3.68	up	**205169_at**	RBBP5	2.94	up
201435_s_at	EIF4E	2.28	up	**209458_x_at**	HBA1 /// HBA2	3.70	up	**202214_s_at**	CUL4B	2.97	up
201153_s_at	MBNL1	2.35	up	**203915_at**	CXCL9	3.72	up	**202181_at**	SUSD6	2.98	up
201455_s_at	NPEPPS	2.37	up	**203887_s_at**	THBD	3.80	up	**205032_at**	ITGA2	3.02	up
201372_s_at	CUL3	2.38	up	**207137_at**	TONSL	3.94	up	**205081_at**	CRIP1	3.05	up
201427_s_at	SEPP1	2.42	up	**209354_at**	TNFRSF14	3.96	up	**203124_s_at**	SLC11A2	3.07	up
221381_s_at	MORF4L1	2.42	up	**205577_at**	PYGM	3.98	up	**209037_s_at**	EHD1	3.09	up
1405_i_at	CCL5	2.42	up	**209429_x_at**	EIF2B4	4.04	up	**203125_x_at**	SLC11A2	3.15	up
221540_x_at	GTF2H2 /// GTF2H2B /// GTF2H2C /// GTF2H2C_2	2.48	up	**202702_at**	TRIM26	4.10	up	**203060_s_at**	PAPSS2	3.22	up
220868_s_at	SLC7A10	2.55	up	**207133_x_at**	ALPK1	4.25	up	**202213_s_at**	CUL4B	3.24	up
221170_at	HRH4	2.62	up	**203845_at**	KAT2B	4.25	up	**203045_at**	NINJ1	3.33	up
201319_at	MYL12A	2.64	up	**207196_s_at**	TNIP1	4.29	up	**209035_at**	MDK	3.35	up
201384_s_at	NBR1	2.65	up	**207163_s_at**	AKT1	4.31	up	**205082_s_at**	AOX1	3.38	up
38710_at	OTUB1	2.66	up	**207175_at**	ADIPOQ	4.33	up	**215485_s_at**	ICAM1	3.41	up
220707_s_at	FOXRED2	2.74	up	**117_at**	HSPA6	4.54	up	**205179_s_at**	ADAM8	3.42	up
203684_s_at	BCL2	2.75	up	**205579_at**	HRH1	4.56	up	**203062_s_at**	1.00 MDC	3.47	up
203725_at	GADD45 A	2.79	up	**202662_s_at**	ITPR2	4.57	up	**209018_s_at**	PINK1	3.51	up
200893_at	TRA2B	2.83	up	**209341_s_at**	IKBKB	4.63	up	**205127_at**	PTGS1	3.55	up
201370_s_at	CUL3	2.87	up	**203879_at**	PIK3CD	4.73	up	**209019_s_at**	PINK1	3.57	up

200006_at	PARK7	2.92	up	**209431_s_at**	PATZ1	4.77	up	**203059_s_at**	PAPSS2	3.63	up
201101_s_at	BCLAF1	2.99	up	**202643_s_at**	TNFAIP3	4.79	up	**203058_s_at**	PAPSS2	3.84	up
200956_s_at	SSRP1	3.05	up	**202661_at**	ITPR2	4.81	up	**205091_x_at**	RECQL	3.84	up
201109_s_at	THBS1	3.05	up	**207188_at**	CDK3 /// TEN1-CDK3	4.82	up	**209021_x_at**	ATG13	3.88	up
200064_at	HSP90AB 1	3.08	up	**203836_s_at**	MAP3K5	4.84	up	**209039_x_at**	EHD1	3.94	up
220651_s_at	MCM10	3.11	up	**209390_at**	TSC1	4.87	up	**203142_s_at**	AP3B1	3.97	up
201436_at	EIF4E	3.12	up	**209455_at**	FBXW11	4.94	up	**203123_s_at**	SLC11A2	4.02	up
203602_s_at	ZBTB17	3.15	up	**205557_at**	BPI	4.98	up	**205041_s_at**	ORM1 /// ORM2	4.05	up
200920_s_at	BTG1	3.16	up	**205581_s_at**	NOS3	5.11	up	**205045_at**	AKAP10	4.15	up
203659_s_at	TRIM13	3.19	up	**203870_at**	USP46	5.27	up	**203063_at**	PPM1F	4.15	up
221125_s_at	KCNMB3	3.20	up	**209389_x_at**	DBI	5.34	up	**205060_at**	PARG	4.22	up
200955_at	IMMT	3.22	up	**203921_at**	CHST2	5.44	up	**201423_s_at**	CUL4A	4.41	up
200862_at	DHCR24	3.34	up	**209327_s_at**	NOP16	5.70	up	**205087_at**	RWDD3	4.69	up
201006_at	PRDX2	3.41	up	**203872_at**	ACTA1	5.71	up	**202188_at**	NUP93	4.77	up
200757_s_at	CALU	3.43	up	**209323_at**	PRKRIR	5.76	up	**203037_s_at**	MTSS1	4.84	up
37793_r_at	RAD51D	3.48	up	**207158_at**	APOBEC1	5.81	up	**202176_at**	ERCC3	4.86	up
201495_x_at	MYH11	3.73	up	**207134_x_at**	TPSAB1 /// TPSB2	5.92	up	**203036_s_at**	MTSS1	5.28	up
41220_at	SEPT9	3.73	up	**207145_at**	MSTN	5.93	up	**205180_s_at**	ADAM8	5.48	up
203720_s_at	ERCC1	3.94	up	**203828_s_at**	IL32	5.93	up	**203028_s_at**	CYBA	5.57	up
201461_s_at	MAPKAP K2	3.95	up	**203809_s_at**	AKT2	6.03	up	**209038_s_at**	EHD1	5.79	up
201460_at	MAPKAP K2	4.02	up	**205586_x_at**	VGF	6.15	up	**205023_at**	RAD51	6.17	up
64440_at	IL17RC	4.06	up	**203917_at**	CXADR	6.31	up	**205024_s_at**	RAD51	6.70	up
211506_s_at	CXCL8	4.08	up	**203843_at**	RPS6KA3	6.36	up	**203042_at**	LAMP2	7.34	up

202859_x_at	CXCL8	4.11	up
201496_x_at	MYH11	4.12	up
201310_s_at	NREP	4.13	up
201044_x_at	DUSP1	4.27	up
203655_at	XRCC1	4.27	up
203648_at	TATDN2	4.29	up
221627_at	TRIM10	4.32	up
203786_s_at	TPD52L1	4.37	up
200997_at	RBM14-RBM4 /// RBM4	4.40	up
200851_s_at	IST1	4.52	up
38290_at	RGS14	4.73	up
201058_s_at	MYL9	4.83	up
37012_at	CAPZB	4.87	up
823_at	CX3CL1	4.87	up
201064_s_at	PABPC4	4.97	up
203601_s_at	ZBTB17	5.01	up
201041_s_at	DUSP1	5.27	up
203617_x_at	ELK1	5.32	up
201083_s_at	BCLAF1	5.38	up
200806_s_at	HSPD1	5.39	up
200787_s_at	PEA15	5.40	up
200667_at	UBE2D3	5.47	up
220630_s_at	CHIA	5.47	up
200640_at	YWHAZ	5.48	up
222343_at	BCL2L11	5.50	up
221097_s_at	KCNMB2	5.50	up
**203811_s_at**	DNAJB4	6.59	up
**203925_at**	GCLM	6.68	up
**209337_at**	PSIP1	6.80	up
**203857_s_at**	PDIA5	6.84	up
**203854_at**	CFI	6.91	up
**205576_at**	SERPIND1	7.04	up
**205572_at**	ANGPT2	7.08	up
**202666_s_at**	ACTL6A	7.21	up
**202696_at**	OXSR1	7.38	up
**202670_at**	MAP2K1	7.39	up
**203810_at**	DNAJB4	7.64	up
**209344_at**	TPM4	7.94	up
**203923_s_at**	CYBB	8.32	up
**209395_at**	CHI3L1	8.39	up
**209322_s_at**	SH2B1	9.16	up
**208075_s_at**	CCL7	9.41	up
**209342_s_at**	IKBKB	9.62	up
**205580_s_at**	HRH1	11.03	up
216248_s_at	NR4A2	22.89	up
**204622_x_at**	NR4A2	23.69	up
**204621_s_at**	NR4A2	24.11023	up
**207123_s_at**	MATN4	7-.12659	up
**203141_s_at**	AP3B1	9.47	up
**205027_s_at**	MAP3K8	14.11	up

221342_at	C6orf25	5.56	up
200601_at	ACTN4	5.58	up
36004_at	IKBKG	5.59	up
200799_at	HSPA1A /// HSPA1B	5.61	up
221563_at	DUSP10	5.63	up
35617_at	MAPK7	5.63	up
32029_at	PDPK1	5.64	up
221690_s_at	NLRP2	5.67	up
32699_s_at	PVR	5.68	up
200639_s_at	YWHAZ	5.70	up
200878_at	EPAS1	5.75	up
32128_at	CCL18	5.79	up
200788_s_at	PEA15	5.79	up
200602_at	APP	5.80	up
200632_s_at	NDRG1	5.82	up
200609_s_at	WDR1	5.83	up
221583_s_at	KCNMA1	5.89	up
220626_at	SERPINA 10	5.93	up
200756_x_at	CALU	5.93	up
200743_s_at	TPP1	5.97	up
200957_s_at	SSRP1	5.99	up
221284_s_at	SRC	6.01	up
221584_s_at	KCNMA1	6.04	up
200621_at	CSRP1	6.05	up
203664_s_at	POLR2D	6.06	up

203666_at	CXCL12	6.09	up
201490_s_at	PPIF	6.11	up
221049_s_at	POLL	6.11	up
36019_at	STK19	6.24	up
220558_x_at	TSPAN32	6.32	up
220955_x_at	RAB23	6.37	up
201108_s_at	THBS1	6.40	up
221253_s_at	BLOC1S5-TXNDC5 /// TXNDC5	6.42	up
221232_s_at	ANKRD2	6.43	up
220956_s_at	EGLN2 /// RAB4B-EGLN2	6.45	up
200965_s_at	ABLIM1	6.49	up
201146_at	NFE2L2	6.52	up
201499_s_at	USP7	6.54	up
201493_s_at	PUM2	6.79	up
222242_s_at	KLK5	6.79	up
221691_x_at	NPM1	6.82	up
200811_at	CIRBP	6.86	up
200658_s_at	PHB	7.02	up
201069_at	MMP2	7.63	up
220890_s_at	APOLD1 /// DDX47	7.78	up
221824_s_at	MARCH8	7.92	up
203685_at	BCL2	8.20	up
201143_s_at	EIF2S1	8.73	up

201119_s_at	COX8A	8.87	up
221679_s_at	ABHD6	8.91	up
201125_s_at	ITGB5	9.24	up
221898_at	PDPN	9.58	up

(up)—overexpression in comparison to the control; (down)—downregulated in comparison to the control;

C—control; H_12. H_24. H_48—periods of exposure to cisplatin
