# Supplementary material for: Expression Profile of mRNAs and miRNAs Related to the Oxidative-Stress Phenomenon in the Ishikawa Cell Line Treated Either Cisplatin or Salinomycin
Source: Biomedicines. 2022 May 20;10(5):1190. doi: 10.3390/biomedicines10051190 (PMC9138494; doi:10.3390/biomedicines10051190)
Supplement: Supplementary file 1 [file biomedicines-10-01190-s001.zip › table s2.pdf]

Table S2. List of all the mRNAs (significantly differentiated; $p < 0.05$) cell culture treated with salinomycin in comparison to a control culture.

H_12 vs C				H_24 vs C				H_48 vs C			
ID	Gene	Fold Change	up/down	ID	Gene	Fold Change	up/down	ID	Gene	Fold Change	up/down
**211688_x_at**	KIR3DL1 /// KIR3DL2	-9.30	down	**205937_at**	CGREF1	-17.89	down	**207746_at**	POLQ	-8.64	down
**212006_at**	UBXN4	-8.79	down	**205945_at**	IL6R	-17.13	down	**207542_s_at**	AQP1	-6.62	down
**202535_at**	FADD	-8.51	down	**205938_at**	PPM1E	-16.89	down	**207774_at**	ATG10	-6.61	down
**211828_s_at**	TNIK	-7.76	down	**205916_at**	S100A7	-9.12	down	**207739_s_at**	GAGE1 /// GAGE12D /// GAGE12F /// GAGE12G /// GAGE12I /// GAGE12J /// GAGE13 /// GAGE2A ///	-6.04	down

									GAGE2B /// GAGE2C /// GAGE2D /// GAGE2E /// GAGE3 /// GAGE4 /// GAGE5 /// GAGE6 /// GAGE7 /// GAGE8		
**202435_s_at**	CYP1B1	-7.13	down	**205905_s_at**	MICA /// MICB	-7.70	down	**207952_at**	IL5	-5.93	down
**202467_s_at**	COPS2	-6.23	down	**205672_at**	XPA	-6.79	down	**207727_s_at**	MUTYH	-5.89	down
**202513_s_at**	PPP2R5D	-6.21	down	**207574_s_at**	GADD45B	-6.13	down	**210372_s_at**	TPD52L1	-5.88	down
**211607_x_at**	EGFR	-6.15	down	**207810_at**	F13B	-5.94	down	**207957_s_at**	PRKCB	-5.69	down

**202503_s_at**	KIAA0101	-6.15	down	**207643_s_at**	TNFRSF1A	-5.66	down	**207966_s_at**	GLG1	-5.69	down
**211883_x_at**	CEACAM1	-5.93	down	**205904_at**	MICA	-5.60	down	**207940_x_at**	CNR1	-5.68	down
**202451_at**	GTF2H1	-5.51	down	**205919_at**	HBE1	-4.88	down	**207723_s_at**	KLRC3	-5.62	down
**212008_at**	UBXN4	-5.37	down	**205681_at**	BCL2A1	-4.53	down	**207769_s_at**	PQBP1	-5.60	down
**206049_at**	SELP	-5.18	down	**221271_at**	IL21	-4.47	down	**207967_at**	VPS45	-5.60	down
**211687_x_at**	KIR3DL1	-4.89	down	**205683_x_at**	TPSAB1 /// TPSB2	-4.29	down	**207714_s_at**	SERPINH1	-5.55	down
**206044_s_at**	BRAF /// KIAA1549	-4.72	down	**207607_at**	ASCL2	-4.23	down	**207968_s_at**	MEF2C	-5.41	down
**202505_at**	SNRPB2	-4.54	down	**205729_at**	OSMR	-4.15	down	**207979_s_at**	CD8B	-5.26	down
**211692_s_at**	BBC3	-4.36	down	**207608_x_at**	CYP1A2	-4.11	down	**207964_x_at**	IFNA4	-4.69	down
**211611_s_at**	ATF6B /// TNXB	-4.13	down	**205685_at**	CD86	-3.99	down	**210288_at**	KLRG1	-4.53	down

**206051_at**	ELAVL4	-4.06	down	**205885_s_at**	ITGA4	-3.68	down	**207741_x_at**	TPSAB1	-4.32	down
**202466_at**	PAPD7	-4.06	down	**207626_s_at**	SLC7A2	-3.66	down	**210373_at**	NPRL2	-3.50	down
**206064_s_at**	PPIL2	-3.82	down	**205892_s_at**	FABP1	-3.65	down	**207974_s_at**	SKP1	-3.48	down
**211831_s_at**	THPO	-3.65	down	**207629_s_at**	ARHGEF2	-3.53	down	**210244_at**	CAMP	-3.44	down
**202437_s_at**	CYP1B1	-3.47	down	**205707_at**	IL17RA	-3.51	down	**210374_x_at**	PTGER3	-3.39	down
**211695_x_at**	MUC1	-3.45	down	**207617_at**	DDX3X	-3.35	down	**210271_at**	NEUROD2	-3.33	down
**202436_s_at**	CYP1B1	-3.43	down	**205708_s_at**	TRPM2	-3.22	down	**210250_x_at**	ADSL	-3.19	down
**211889_x_at**	CEACAM1	-3.36	down	**205900_at**	KRT1	-3.16	down	**210241_s_at**	TP53TG1	-3.16	down
**211869_at**	---	-3.34	down	**205692_s_at**	CD38	-3.12	down	**210255_at**	RAD51B	-2.78	down
**206060_s_at**	PTPN22	-3.21	down	**205686_s_at**	CD86	-2.99	down	**210284_s_at**	TAB2	-2.74	down
**212007_at**	UBXN4	-3.19	down	**207804_s_at**	FCN2	-2.91	down	**207548_at**	GRM7	-2.72	down

**211890_x_at**	CAPN3	-3.16	down	**205898_at**	CX3CR1	-2.83	down	**210260_s_at**	TNFAIP8	-2.42	down
**202434_s_at**	CYP1B1	-3.13	down	**207598_x_at**	XRCC2	-2.75	down	**207709_at**	PRKAA2	-2.42	down
**211897_s_at**	CRHR1 /// MGC57346-CRHR1	-3.12	down	**207645_s_at**	CHD1L	-2.71	down	**210362_x_at**	PML	-2.34	down
**211893_x_at**	CD6	-2.98	down	**207808_s_at**	PROS1	-2.57	down	**210367_s_at**	PTGES	-2.34	down
**202455_at**	HDAC5	-2.97	down	**205887_x_at**	MSH3	-2.42	down	**210360_s_at**	MTSS1	-2.10	down
**202549_at**	VAPB	-2.97	down	**207614_s_at**	CUL1	-2.28	down	**210257_x_at**	CUL4B	-2.08	down
**202545_at**	PRKCD	-2.95	down	**205891_at**	ADORA2B	-2.27	down	**210242_x_at**	ST20	-2.07	down
**202457_s_at**	PPP3CA	-2.88	down	**205928_at**	ZNF443	-2.11	down	**208046_at**	HIST1H4A	2.02	up
**211605_s_at**	RARA	-2.67	down	**207616_s_at**	TANK	-2.08	down	**207668_x_at**	PDIA6	2.14	up
**212009_s_at**	STIP1	-2.64	down	**205859_at**	LY86	1.74	up	**207686_s_at**	CASP8	2.18	up

**206063_x_at**	PPIL2	-2.59	down	**205838_at**	GYPA	2.02	up	**210236_at**	PPFIA1	2.23	up
**202530_at**	MAPK14	-2.56	down	**207844_at**	IL13	2.04	up	**207691_x_at**	ENTPD1	2.25	up
**206054_at**	KNG1	-2.53	down	**207857_at**	LILRA2	2.07	up	**210359_at**	MTSS1	2.25	up
**206057_x_at**	LOC105369261 /// SPN	-2.52	down	**207814_at**	DEFA6	2.08	up	**207655_s_at**	BLNK	2.38	up
**202550_s_at**	VAPB	-2.50	down	**205858_at**	NGFR	2.11	up	**210235_s_at**	PPFIA1	2.53	up
**211900_x_at**	CD6	-2.48	down	**207566_at**	MR1	2.14	up	**210383_at**	SCN1A	2.54	up
**202453_s_at**	GTF2H1	-2.27	down	**205866_at**	FCN3	2.22	up	**210339_s_at**	KLK2	2.61	up
**206056_x_at**	LOC105369261 /// SPN	-2.02	down	**207815_at**	PF4V1	2.22	up	**210309_at**	RECQL5	2.85	up
**211610_at**	KLF6	-1.90	down	**205774_at**	F12	2.39	up	**210240_s_at**	CDKN2D	2.92	up
**202500_at**	DNAJB2	-1.85	down	**205770_at**	GSR	2.41	up	202859_x_at	CXCL8	3.00	up
**211744_s_at**	CD58	1.82	up	**205837_s_at**	GYPA	2.48	up	**208038_at**	IL1RL2	3.05	up

**211763_s_at**	UBE2B	2.03	up	**207817_at**	IFNW1	2.57	up	**207902_at**	IL5RA	3.09	up
**211804_s_at**	CDK2	2.06	up	**207613_s_at**	CAMK2A	2.59	up	**207901_at**	IL12B	3.10	up
**202637_s_at**	ICAM1	2.06	up	**207609_s_at**	CYP1A2	2.64	up	211506_s_at	CXCL8	3.21	up
**202607_at**	NDST1	2.11	up	**205630_at**	CRH	2.76	up	**210379_s_at**	TLK1	3.22	up
**206026_s_at**	TNFAIP6	2.14	up	**205624_at**	CPA3	2.83	up	**208022_s_at**	CDC14B	3.23	up
**206025_s_at**	TNFAIP6	2.21	up	**205863_at**	S100A12	2.83	up	**207795_s_at**	KLRD1	3.39	up
**202558_s_at**	HSPA13	2.22	up	**207571_x_at**	THEMIS2	2.88	up	**208010_s_at**	PTPN22	3.51	up
**202450_s_at**	CTSK	2.27	up	**207840_at**	CD160	2.93	up	**210208_x_at**	BAG6	3.59	up
**202606_s_at**	TLK1	2.31	up	**205620_at**	F10	2.97	up	**207995_s_at**	CLEC4M	3.59	up
**211820_x_at**	GYPA	2.36	up	**205650_s_at**	FGA	2.98	up	**208023_at**	TNFRSF4	3.71	up
**211788_s_at**	TREX2	2.39	up	**205647_at**	RAD52	3.07	up	**207788_s_at**	SORBS3	3.87	up

**202555_s_at**	MYLK	2.40	up	**207821_s_at**	PTK2	3.09	up	**208018_s_at**	HCK	4.07	up
**211732_x_at**	HNMT	2.41	up	**205841_at**	JAK2	3.09	up	**208003_s_at**	NFAT5	4.08	up
**211945_s_at**	ITGB1	2.42	up	**205749_at**	CYP1A1	3.15	up	**210342_s_at**	TPO	4.31	up
**211624_s_at**	DRD2	2.49	up	**207827_x_at**	SNCA	3.15	up	**208026_at**	HIST1H4F	4.50	up
**202608_s_at**	NDST1	2.50	up	**205654_at**	C4BPA	3.22	up	**210211_s_at**	HSP90AA1	4.68	up
**211711_s_at**	PTEN	2.51	up	**205667_at**	WRN	3.39	up	**207554_x_at**	TBXA2R	5.18	up
**202617_s_at**	IRAK1 /// MECP2	2.51	up	**205786_s_at**	ITGAM	3.42	up	**207994_s_at**	OPRM1	5.33	up
**202444_s_at**	ERLIN1	2.54	up	**207565_s_at**	MR1	3.42	up	**208028_s_at**	GPX5	5.34	up
**211652_s_at**	LBP	2.66	up	**205746_s_at**	ADAM17	3.44	up	**207989_at**	OPRM1	5.41	up
**211783_s_at**	MTA1	2.70	up	**205819_at**	MARCO	3.44	up	**207798_s_at**	ATXN2L	5.42	up
**211696_x_at**	HBB	2.75	up	**205750_at**	BPHL	3.47	up	**207872_s_at**	LILRA1	5.43	up

**202441_at**	ERLIN1	2.84	up	**205745_x_at**	ADAM17	3.64	up	**210198_s_at**	PLP1	5.46	up
**211658_at**	PRDX2	2.89	up	**205867_at**	PTPN11	3.65	up	**207890_s_at**	MMP25	5.47	up
**211657_at**	CEACAM6	2.90	up	**205640_at**	ALDH3B1	3.71	up	**207892_at**	CD40LG	5.59	up
**211762_s_at**	KPNA2	2.91	up	**208075_s_at**	CCL7	3.75	up	**207802_at**	CRISP3	5.60	up
**211928_at**	DYNC1H1	3.07	up	**207847_s_at**	MUC1	3.76	up	**207932_at**	IFNA8	5.61	up
**211728_s_at**	HYAL3	3.15	up	**205666_at**	FMO1	3.77	up	**207919_at**	ART1	5.61	up
**202556_s_at**	MCRS1	3.17	up	**205623_at**	ALDH3A1	3.77	up	**207860_at**	NCR1	5.62	up
**211699_x_at**	HBA1 /// HBA2	3.17	up	**205621_at**	ALKBH1	3.78	up	**200833_s_at**	RAP1B	5.65	up
**202553_s_at**	SYF2	3.19	up	**205844_at**	1.00 VNN	3.82	up	**207980_s_at**	CITED2	5.67	up
**207528_s_at**	SLC7A11	3.25	up	**205649_s_at**	FGA	3.82	up	**207861_at**	CCL22	5.68	up
**211706_s_at**	CDK19	3.33	up	**205668_at**	LY75	3.88	up	**210376_x_at**	ELK1	5.73	up

**202638_s_at**	ICAM1	3.38	up	**205827_at**	CCK	3.88	up	**207926_at**	GP5	5.84	up
**211719_x_at**	FN1	3.41	up	**205743_at**	STAC	3.99	up	**207900_at**	CCL17	5.95	up
209921_at	SLC7A11	3.41	up	**205766_at**	TCAP	4.02	up	**210233_at**	IL1RAP	6.15	up
**202616_s_at**	MECP2	3.45	up	**205753_at**	CRP	4.02	up	**208000_at**	GML	6.35	up
**211661_x_at**	PTAFR	3.48	up	**205639_at**	AOAH	4.03	up	**208034_s_at**	PROZ	6.47	up
**211851_x_at**	BRCA1	3.50	up	**205660_at**	OASL	4.08	up	**210375_at**	PTGER3	6.51	up
**202557_at**	HSPA13	3.51	up	**205884_at**	ITGA4	4.22	up	**207796_x_at**	KLRD1	6.57	up
**211956_s_at**	EIF1	3.52	up	**205842_s_at**	JAK2	4.25	up	**207539_s_at**	IL4	6.79	up
**211741_x_at**	PSG3	3.61	up	**205782_at**	FGF7	4.31	up	**210216_x_at**	RAD1	7.05	up
**211805_s_at**	SLC8A1	3.65	up	**207610_s_at**	ADGRE2	4.34	up	**207777_s_at**	SP140	7.27	up
**202618_s_at**	MECP2	3.74	up	**205779_at**	RAMP2	4.42	up	**207550_at**	MPL	8.16	up

**2118<br>03_a<br>t**	CDK2	3.75	up	**2056<br>51_x<br>_at**	RAPGEF<br>4	4.42	up	**210224_at**	MR1	8.32	up
**2119<br>26_s<br>_at**	MYH9	3.79	up	**2078<br>50_at**	CXCL3	4.44	up	**207782_s_a<br>t**	PSEN1	9.25	up
**2117<br>96_s<br>_at**	TRBC1<br>///<br>TRBC2<br>///<br>TRBV19<br>///<br>TRBV3-<br>1 ///<br>TRBV5-<br>4 ///<br>TRBV6-<br>5 ///<br>TRBV7-<br>2	3.79	up	**2058<br>20_s<br>_at**	APOC3	4.55	up	**210223_s_a<br>t**	MR1	9.50	up
**2025<br>81_a<br>t**	HSPA1<br>A ///<br>HSPA1<br>B	3.81	up	**2057<br>54_at**	F2	4.55	up	**207783_x_a<br>t**	HUWE<br>1	13.03	up
**2117<br>34_s<br>_at**	FCER1<br>A	3.86	up	**2057<br>30_s<br>_at**	ABLIM3	4.59	up	**207555_s_a<br>t**	TBXA2<br>R	13.95	up
**2119<br>05_s<br>_at**	ITGB4	3.87	up	**2058<br>80_at**	PRKD1	4.72	up	**207536_s_a<br>t**	TNFRS<br>F9	15.23	up
**2119<br>03_s<br>_at**	MPL	3.90	up	**2078<br>16_at**	LALBA	4.76	up	**204621_s_a<br>t**	NR4A2	15.45	up

**211978_x_at**	PPIA	3.91	up	**205664_at**	KIN	4.90	up	**207538_at**	IL4	15.82	up
**215485_s_at**	ICAM1	3.99	up	**205756_s_at**	F8	5.04	up	**202859_x_at**	CXCL8	15.88	up
**211865_s_at**	FZR1	4.06	up	**205760_s_at**	OGG1	5.11	up	**207535_s_at**	NFKB2	15.94	up
**211936_at**	HSPA5	4.08	up	**207557_s_at**	RYR2	5.12	up	**204622_x_at**	NR4A2	15.96	up
**211968_s_at**	HSP90AA1	4.09	up	**207852_at**	CXCL5	5.31	up	216248_s_at	NR4A2	16.01	up
**202620_s_at**	PLOD2	4.12	up	**205824_at**	HSPB2	5.35	up				
**211852_s_at**	ATRN	4.12	up	**207858_s_at**	PKLR	5.42	up				
**206039_at**	RAB33A	4.14	up	**205870_at**	BDKRB2	5.64	up				
**211767_at**	GINS4	4.16	up	**205815_at**	REG3A	5.80	up				
**211821_x_at**	GYPA	4.21	up	**205733_at**	BLM	6.06	up				
**211724_x_at**	MIOS	4.37	up	**205857_at**	SLC18A2	6.15	up				

**211786_at**	TNFRSF9	4.37	up	**205879_x_at**	RET	6.42	up
**211764_s_at**	UBE2D1	4.42	up	**205831_at**	CD2	6.99	up
**211743_s_at**	PRG2	4.46	up	**205778_at**	KLK7	7.03	up
**211668_s_at**	PLAU	4.53	up	**207556_s_at**	DGKZ	7.05	up
**211856_x_at**	CD28	4.57	up	**205629_s_at**	CRH	8.04	up
**211745_x_at**	HBA1 /// HBA2	4.78	up	**205821_at**	KLRC4-KLRK1 /// KLRK1	9.29	up
**211841_s_at**	TNFRSF25	4.89	up	**205970_at**	MT3	9.53	up
**202619_s_at**	PLOD2	4.93	up	**205967_at**	HIST1H4C	10.02	up
**211819_s_at**	SORBS1	4.96	up	**205868_s_at**	PTPN11	10.37	up
**211969_at**	HSP90AA1	5.01	up	**205988_at**	CD84	12.03	up

**211833_s_at**	BAX	5.08	up	**205992_s_at**	IL15	13.11	up
**211922_s_at**	CAT	5.09	up	**205977_s_at**	EPHA1	14.20	up
**211909_x_at**	PTGER3	5.20	up	**206001_at**	NPY	14.31	up
**211766_s_at**	PNLIPRP2	5.35	up	**206009_at**	ITGA9	14.66	up
**211919_s_at**	CXCR4	5.39	up	**205962_at**	PAK2	16.04	up
**211630_s_at**	GSS	5.44	up	**206011_at**	CASP1	16.20	up
**206040_s_at**	MAPK11	6.00	up	**205978_at**	KL	16.28	up
**211631_x_at**	B4GALT1	6.02	up	**205984_at**	CRHBP	16.45	up
**202614_at**	SLC30A9	6.15	up	**205961_s_at**	PSIP1	16.50	up
**211769_x_at**	SERINC3	6.15	up	**205960_at**	PDK4	16.67	up
**211676_s_at**	IFNGR1	6.85	up	**205959_at**	MMP13	16.78	up

**211823_s_at**	PXN	7.02	up
**211861_x_at**	CD28	7.28	up
**211839_s_at**	CSF1	7.35	up
**211713_x_at**	KIAA0101	7.38	up
**211864_s_at**	MYOF	7.60	up
**211705_s_at**	SORBS1	7.93	up
**211765_x_at**	PPIA	8.42	up
**211920_at**	CFB	11.18	up
**211904_x_at**	RAD52	12.54	up
**206020_at**	SOCS6	16.84	up
**206012_at**	LEFTY2	17.66	up
**205027_s_at**	MAP3K8	27.14	up

(up)—overexpression in comparison to the control; (down)—downregulated in comparison to the control;

C—control; H_12. H_24. H_48—periods of exposure to salinomycin
